# Predischarge blood count and nutritional risk marker patterns associated with 30-day unplanned readmissions for hematological toxicity after radiotherapy for nasopharyngeal carcinoma: a multicenter cohort study

**DOI:** 10.3389/fmed.2026.1929774

**Published:** 2026-10-14

**Authors:** Guifei Zhou, Qianxi Sha, Lihong Zhao, Suren Rao Sooranna, Ying Wang, Huiying Tan

**Affiliations:** 1 School of Nursing, Youjiang Medical University for Nationalities, Baise, Guangxi, China; 2 Department of Oncology, People's Hospital of Qianxinan Prefecture, Guizhou Province, China; 3 Faculty of Medicine, Imperial College London, London, United Kingdom; 4 Cardiac Intensive Care Unit, Department of Cardiology, Affiliated Hospital of Youjiang Medical University for Nationalities, Baise, Guangxi, China

**Keywords:** association rule mining, discharge transition, Firth logistic regression, hematological toxicity, hospital readmission, nasopharyngeal carcinoma, radiotherapy, supportive care

## Abstract

**Background:**

Early unplanned readmissions after radiotherapy-based treatment may reflect residual toxicity and gaps in supportive care coordination. We examined associations between predischarge hematological and albumin marker patterns and 30-day hematological toxicity-related unplanned readmission in nasopharyngeal carcinoma (NPC).

**Methods:**

This multicenter retrospective cohort study included 607 patients discharged after hospitalization involving radiotherapy-based treatment at three tertiary hospitals in southwestern China between December 2018 and March 2026. The primary complete-case sample comprised 588 patients with predischarge absolute neutrophil count (ANC), platelet count (PLT), and albumin measurements. Core markers were ANC <1.5 × 10⁹/L, PLT <200 × 10⁹/L, and albumin <35 g/L. We used association rule mining and Firth penalized logistic regression. Chemotherapy and supportive care variables were examined in sensitivity analyses, and all-cause 30-day unplanned readmission was a supportive secondary outcome.

**Results:**

Based on the chronologically first readmission within 30 days, 77 of 607 patients (12.7%) were classified as having an unplanned readmission; 58 of the 77 (75.3%) were classified as hematological toxicity-related. In the primary sample, hematological readmission rates were 4.9%, 13.1%, and 17.5% for zero, one, and two or more core markers, respectively. Adjusted odds ratios (ORs) were 2.86 [95% confidence interval (CI) 1.50–5.46] and 4.33 (95% CI 1.82–10.30) for one and two or more markers versus zero markers. Corresponding ORs were 2.93 and 4.55 after chemotherapy adjustment and 2.59 and 3.47 after supportive care adjustment. The all-cause analysis showed a similar gradient (OR 2.65, 95% CI 1.52–4.61; OR 3.18, 95% CI 1.43–7.05).

**Conclusion:**

Routine predischarge measures were associated with early readmission risk, but the findings do not establish causality or a clinical decision rule. Prospective external validation is required before clinical use.

## Introduction

Hospital readmissions soon after discharge are widely used as a transition-of-care and health-system performance outcome. Although not all readmissions are preventable, early unplanned readmissions may indicate unresolved illness burden, residual treatment toxicity, incomplete symptom control, or insufficient postdischarge coordination. Risk stratification may help target transitional care interventions to patients who need timely follow-up without implying that all readmissions are avoidable (1–3).

Assessing readmission in cancer care is challenging. Patients with cancer may return to the hospital because of disease progression, planned treatment continuation, or acute treatment-related complications. Cancer-specific readmission measures therefore distinguish unplanned urgent or emergency admissions from planned admissions for treatment continuation (4). Prior oncology and head and neck cancer studies have examined rates, causes, and predictors of early readmission or potentially avoidable hospitalization (5–12). However, broad all-cause models may offer limited guidance for targeted supportive care.

Nasopharyngeal carcinoma (NPC) is a distinct head and neck malignancy characterized by marked geographic variation and radiotherapy-centered treatment pathways (13–16). Radiotherapy is often combined with platinum-based chemotherapy, induction chemotherapy, or adjuvant systemic treatment for appropriate risk groups (17–20). During and after radiotherapy or chemoradiotherapy, patients may experience mucositis, nutritional deterioration, increased infection risk, anemia, leukopenia, neutropenia, thrombocytopenia, and other toxicities that can persist beyond the initial hospitalization (21–26). These residual toxicities are clinically relevant at discharge because routine laboratory measurements may indicate the need for further evaluation.

Discharge risk is rarely captured by a single measurement. In this study, predischarge risk markers were defined using routinely available absolute neutrophil count (ANC), platelet count (PLT), and albumin measurements obtained during the 7-calendar-day window ending on and including the index discharge. Association rule mining can identify clear co-occurrence patterns among categorical clinical features, whereas penalized regression can quantify their associations and uncertainty. These established clinical measures were not considered novel biomarkers; the aim was to examine whether their joint patterns provided prognostic information at the transition from inpatient treatment to discharge.

Using a multicenter cohort of patients with NPC discharged after radiotherapy-based treatment, we aimed to describe the spectrum of causes for 30-day unplanned readmissions and identify predischarge hematological and albumin risk marker patterns associated with hematological toxicity-related unplanned readmission. We also evaluated treatment and supportive care confounding, all-cause unplanned readmission as a broader supportive outcome, and the incremental internal performance of simple marker- and rule-based models. Supporting methods and analyses are provided in Supplementary Figures S1–S3 and Tables S1–S20.

## Materials and methods

### Study design and setting

We conducted a multicenter retrospective cohort study across three tertiary hospitals in southwestern China. Patients with NPC were discharged after the first eligible hospitalization involving radiotherapy-based treatment between December 2018 and March 2026. Thirty-day readmissions were identified from the electronic medical record systems of the participating hospitals; readmissions to non-participating hospitals could not be captured. The study used deidentified retrospective data; details of the ethics approval and waiver of informed consent are provided in the Ethics statement. Reporting followed the Strengthening the Reporting of Observational Studies in Epidemiology (STROBE), REporting of studies Conducted using Observational Routinely-collected health Data (RECORD), and Transparent Reporting of a multivariable prediction model for Individual Prognosis Or Diagnosis plus Artificial Intelligence (TRIPOD + AI) recommendations where applicable (27–29).

We evaluated prognostic associations between predischarge risk markers and readmission; causal effects were not estimated. The prespecified adjustment set was used for the primary model. Chemotherapy and supportive care variables were evaluated in secondary sensitivity analyses because treatment categories were sparse and the availability of historical order data differed by center.

### Participants and analytic samples

Patients were eligible if they had documented NPC, were discharged after a hospitalization involving radiotherapy-based treatment during the study period, and had available data on sex, age, discharge date, and 30-day outcomes. Radiotherapy-based treatment included radiotherapy alone, concurrent chemoradiotherapy, radiotherapy after induction chemotherapy, or radiotherapy combined with other systemic treatments recorded in the treatment course. For each patient, the first eligible hospitalization involving radiotherapy-based treatment was defined as the index hospitalization, and its discharge date was defined as the index discharge. Records without a determinable 30-day outcome, those unrelated to the current radiotherapy-based course, and records with insufficient follow-up were excluded. The full cohort included 607 patients. The primary analytic sample included 588 patients with complete ANC, PLT, and albumin values measured during the 7-calendar-day window ending on and including the index discharge; 19 patients with missing core measures were excluded from this complete-case analysis.

### Outcome ascertainment

The primary outcome was hematological toxicity-related unplanned readmission within 30 days after index discharge. Two researchers independently reviewed each identified readmission. For each patient, only the chronologically first readmission within 30 days was used to determine patient-level outcome status. Using the admission purpose, principal readmission diagnosis, and documented reason for that first readmission, the reviewers classified it as planned treatment continuation or unplanned. A first readmission for planned treatment continuation was classified as a non-event; subsequent readmissions did not redefine the outcome. If the first readmission was unplanned, its principal documented reason determined the mutually exclusive readmission category; no severity-based or cause-priority hierarchy was applied. Disagreements at either step were resolved through joint review of the source medical records and consensus. Hematological toxicity-related admissions included myelosuppression, hematological toxicity, or a clinically equivalent cytopenic/toxicity category documented as the principal reason for inpatient admission. Other categories included infection/fever/pulmonary complication, radiation mucositis/pharyngeal symptom, nutritional/gastrointestinal problem, other acute/cardiovascular/cerebrovascular issues, skin/radiation dermatitis, and other/unclear acute causes (Supplementary Table S20). Predischarge ANC, PLT, hemoglobin, albumin, body mass index (BMI), derived marker counts, and rule status were not used to assign the outcome. The analytic file retained one reconciled patient-level 30-day status and, when the first readmission was unplanned, one principal reason.

Only the reconciled consensus classifications were retained; the reviewers’ initial individual classifications were not preserved. Therefore, percentage agreement and Cohen's *κ* could not be calculated. We describe the procedure as independent duplicate review followed by consensus, rather than as blinded adjudication.

### Data harmonization across hospitals

A shared data dictionary was used to harmonize variables. Laboratory units were standardized to × 10⁹/L for blood cell counts and g/L for albumin and hemoglobin. The measurement closest to discharge during the 7-calendar-day window ending on and including the index discharge was selected. Discharge dates, readmission dates, and readmission reasons were checked for internal consistency. Predictor fields and outcome reason fields were stored separately. Potential center effects were evaluated by adding hospital center to Firth models and by excluding the small center with no hematological readmission events.

### Chemotherapy and supportive care variables

Chemotherapy information was reabstracted from executed medication orders and administration records using a shared coding dictionary. Any chemotherapy was coded as present when at least one administration before index discharge was documented. Induction chemotherapy preceded radiotherapy, concurrent chemotherapy was administered during radiotherapy, and predischarge adjuvant chemotherapy followed radiotherapy but preceded index discharge. Internally discordant summary fields were reconciled against dated administration records and documentation of treatment refusal. We recorded regimen group, cumulative administered cycles in the current course through discharge, and days from the most recent chemotherapy administration to discharge. Treatment first administered after index discharge was not considered a baseline exposure and was not modeled as a time-varying exposure during the 30-day outcome window. Only one patient received predischarge adjuvant chemotherapy; this exposure was described but not modeled. Any-chemotherapy and induction indicators were not modeled because 98.3% and 91.8% of the analytic sample, respectively, were exposed, leaving limited unexposed groups. Regimen and platinum categories were summarized rather than modeled because several categories were sparse and strongly aligned with the center. Cumulative drug dose was not consistently recoverable; therefore, documented cycle count was used, and dose was not inferred from regimen names.

Supportive care exposure during the 7-calendar-day window ending on and including index discharge included granulocyte colony-stimulating factor (G-CSF), systemic antimicrobial treatment, nutritional support, red-cell transfusion, platelet transfusion, and human albumin infusion. The window matched the laboratory-measurement window and was intended to characterize care proximal to discharge. For each supportive care variable, a documented administration was coded as 1, no administration in an accessible executed order history was coded as 0, and an inaccessible order history was coded as missing. At Center 3, historical executed order data were inaccessible for 68 early admissions; these variables were coded as missing rather than absent. Red-cell transfusion, platelet transfusion, and albumin infusion were described without multivariable coefficients because exposure counts were very small.

### Predischarge indicators, thresholds, and variable coding

The three core markers were prespecified for the primary analysis: ANC <1.5 × 10⁹/L, PLT <200 × 10⁹/L, and albumin <35 g/L. ANC <1.5 × 10⁹/L corresponds to grade 2 or higher decreased neutrophil count in the Common Terminology Criteria for Adverse Events (CTCAE) v5.0 (22). Albumin <35 g/L is a commonly reported low-albumin boundary in oncology and NPC, but albumin was treated as a non-specific risk marker that may reflect inflammation, illness burden, intake limitation, hydration, or treatment-related catabolism rather than as a diagnosis of malnutrition (23–26, 30). PLT <200 × 10⁹/L has no validated basis as a diagnostic or treatment threshold. It was retained solely as the prespecified exploratory marker for the primary analysis and was not interpreted as thrombocytopenia. We repeated the analysis with PLT <150 × 10⁹/L, a conventional thrombocytopenia boundary (31), and modeled all three measures continuously to assess dependence on categorization (32). No outcome-guided cut-point optimization was performed.

Additional candidate antecedents included hemoglobin (Hb) <100 g/L, BMI <18.5 kg/m^2^, age ≥60 years, sex, stage, and comorbidity. Hb <100 g/L corresponds to grade 2 or higher anemia under CTCAE v5.0 (22), and BMI <18.5 kg/m^2^ is the World Health Organization (WHO) underweight boundary (33). Age ≥60 years was a prespecified rule-mining category and was not treated as a clinical geriatric threshold; regression retained age continuously per 10-year increase. Male sex was coded as 1 and female sex as 0. Comorbidity was coded as 1 when any chronic comorbidity was present and 0 otherwise. Clinical stage was abstracted as recorded in the source record; stage III–IV was coded as 1 and stage I–II as 0, with a separate missing-stage indicator. The core marker count was categorized as 0, 1, or ≥ 2, with 0 as the reference. In continuous sensitivity models, odds ratios (ORs) were scaled per 1 × 10⁹/L lower ANC, 50 × 10⁹/L lower PLT, and 5 g/L lower albumin.

### Association rule mining

Association rule mining fixed hematological toxicity-related unplanned readmission as the consequent (34). Candidate antecedents were categorical fields available before discharge and did not include outcome-defining fields. The prespecified screening thresholds were retained: co-occurrence support ≥1.5%, confidence ≥15%, lift ≥1.5, antecedent length 1–3, and at least nine primary events per retained rule. Co-occurrence support was the proportion of cases with both the antecedent and outcome; confidence was the event rate among patients satisfying the antecedent; lift was confidence divided by the overall event rate. Clinically redundant extensions were pruned in favor of simpler rules. All qualifying rules are provided in the Supplementary Material.

### Statistical analysis and internal performance assessment

Continuous variables were summarized as medians and interquartile ranges (IQRs) and compared using Mann–Whitney *U*-tests. Categorical variables were summarized as counts and percentages and compared using Pearson *χ*^2^ tests or Fisher exact tests, as appropriate. All *P*-values were two-sided, and *P* < 0.05 was considered statistically significant. Firth penalized logistic regression was used for association estimates to reduce small-sample and separation bias (35, 36). Adjusted ORs, profile penalized-likelihood 95% confidence intervals (CIs), and penalized likelihood-ratio *P*-values were reported. The primary risk marker burden model adjusted for age, sex, stage, missing stage, and comorbidity. Rule models used the same adjustment set unless a covariate was part of the antecedent.

To assess chemotherapy-related confounding without replacing the primary model, Model C1 added concurrent chemotherapy and cumulative cycle count. Model C2 was restricted to chemotherapy recipients and added cumulative cycles and the interval from the most recent chemotherapy administration to discharge; concurrent status was omitted from C2 to limit collinearity and parameter burden. A separate analysis was stratified by concurrent-chemotherapy status. For supportive care sensitivity analyses, Model S0 refitted the primary model among patients with evaluable G-CSF, antimicrobial, and nutritional-support data, and Model S1 added those three variables in the same sample. These models were not interpreted as treatment-effect estimates because confounding by indication and temporal proximity to the laboratory measurements remained possible.

For incremental performance, the basic clinical comparator model included age, sex, stage, the missing-stage indicator, and comorbidity; it was not presented as a validated standard clinical model. We compared it with models adding ANC, PLT, or albumin individually on continuous scales; all three continuous measures jointly; all three binary core markers; marker burden strata; or rule count strata. Association ORs were estimated using Firth penalized logistic regression, whereas Brier score, calibration, and decision curve probabilities used Firth's logistic regression with intercept correction (FLIC), which retains the slope coefficients and recalibrates the intercept (37). We reported the area under the receiver operating characteristic curve (AUC), Brier score, calibration-in-the-large, calibration intercept and slope, and decision curve net benefit (38–40). Apparent AUC 95% CIs and paired differences in AUC were estimated from 1,000 patient-level bootstrap resamples. Internal validation involved repeated model fitting in each bootstrap sample, evaluating performance in both the bootstrap and original samples, and subtracting average optimism from apparent AUC, Brier score, and calibration slope (41). Rule discovery and pruning were not repeated in each resample; thus, rule count performance is conditional on the retained rules and may remain optimistic.

Decision curve analysis used threshold probabilities from 0.03 to 0.30 to encompass the observed event rate and a broad range potentially relevant to low-burden follow-up actions. The decision curve analysis was exploratory because no validated intervention threshold exists. Category-based reclassification metrics were not calculated because no externally established risk categories or action thresholds exist, and data-derived categories would imply unsupported clinical boundaries. No random train/test split was used because the limited event count would inefficiently discard information; bootstrap estimates represent internal evaluation only and not external validation.

Primary modeling and internal performance assessment were conducted using R 4.4.2. Data harmonization, sensitivity analyses, and independent verification were performed using Python 3.11.9 with pandas, NumPy, SciPy, scikit-learn, and a documented adjusted-score implementation of Firth regression. A fixed seed of 20251028 was used for resampling.

### Sensitivity analyses

Sensitivity analyses excluded patients whose chronologically first readmission within 30 days was for planned treatment continuation, used PLT <150 × 10⁹/L, retained the laboratory measures continuously, restricted analyses to complete stage data, added center, excluded the zero-event center, and incorporated chemotherapy or supportive care variables as described above. All-cause 30-day unplanned readmission was evaluated as a supportive secondary outcome using the same marker burden categories and adjustment set, without reselecting cut-points or rules. Because this broader endpoint was evaluated in the same cohort and included all hematological events, it was an outcome-definition robustness analysis rather than independent validation.

## Results

### Sample and readmission spectrum

The full cohort included 607 patients. Among them, 77 (12.7%) were classified as having an unplanned readmission based only on the chronologically first readmission within 30 days recorded at the three participating hospitals. Of these, 58 (75.3%) were classified as hematological toxicity/myelosuppression, followed by infection/fever/pulmonary complication (*n* = 6), radiation mucositis/pharyngeal symptom (*n* = 6), nutritional/gastrointestinal problem (*n* = 3), other acute/cardiovascular/cerebrovascular (*n* = 2), skin/radiation dermatitis (*n* = 1), and other/unclear acute cause (*n* = 1; Figure 1). In 38 patients, the chronologically first readmission within 30 days was for planned treatment continuation; these patients were classified as non-events. The three core markers were complete for 588 patients, and all 58 primary events were retained.

**Figure 1:**
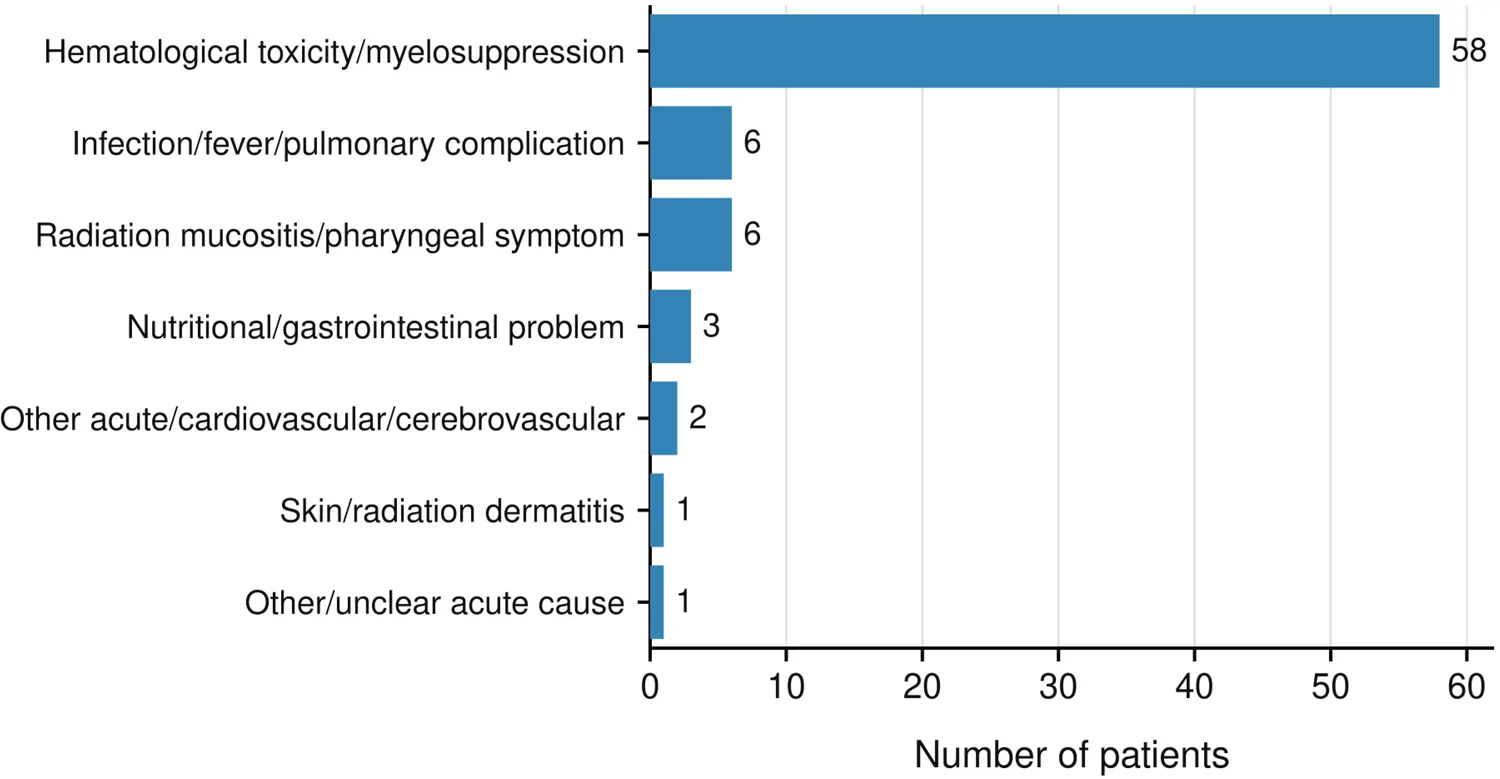
Principal reasons for the chronologically first readmission within 30 days among patients whose first readmission was unplanned (*n* = 77; one count per patient).

### Baseline characteristics

In the 588-patient analytic sample, the median age was 50.0 years, 72.6% were male, 90.1% had stage III–IV disease, and 47.6% had recorded comorbidity. Patients with the primary outcome had lower median PLT (185.0 vs. 208.5 × 10⁹/L; *P* = 0.024) and were more likely to have ANC <1.5 × 10⁹/L (10.3% vs. 3.0%; *P* = 0.015), PLT <200 × 10⁹/L (63.8% vs. 45.1%; *P* = 0.007), and albumin <35 g/L (24.1% vs. 14.0%; *P* = 0.039) (Table 1).

**Table 1:** Baseline characteristics in the primary analytic sample by hematological toxicity-related readmission status.

Variable	Overall (*N* = 588)	No hematological readmission (*N* = 530)	Hematological readmission (*N* = 58)	*P*-value
Age, years	50.0 (43.0, 57.0)	50.0 (43.0, 57.0)	49.0 (42.2, 54.0)	0.461
Length of stay, days	46.0 (40.0, 51.0)	46.0 (40.0, 51.0)	46.5 (39.8, 53.0)	0.242
BMI, kg/m^2^	23.1 (20.9, 25.5)	23.2 (20.9, 25.5)	22.4 (20.5, 24.9)	0.210
Hemoglobin, g/L	111.0 (98.0, 122.0)	111.0 (99.0, 122.0)	108.5 (91.5, 118.0)	0.078
ANC,×10⁹/L	3.74 (2.70, 5.96)	3.73 (2.71, 5.79)	4.00 (2.37, 7.68)	0.838
PLT,×10⁹/L	206.0 (160.0, 261.2)	208.5 (161.0, 265.0)	185.0 (155.2, 231.5)	0.024
Albumin, g/L	39.1 (36.5, 41.7)	39.1 (36.7, 41.8)	39.2 (35.4, 41.4)	0.294
Age ≥ 60 years	94 (16.0)	88 (16.6)	6 (10.3)	0.217
Male sex	427 (72.6)	388 (73.2)	39 (67.2)	0.333
Stage III–IV	530 (90.1)	479 (90.4)	51 (87.9)	0.553
Missing stage	10 (1.7)	9 (1.7)	1 (1.7)	1.000
Comorbidity present	280 (47.6)	252 (47.5)	28 (48.3)	0.916
BMI <18.5 kg/m^2^	35 (6.0)	29 (5.5)	6 (10.5)	0.138
Hemoglobin <100 g/L	160 (27.2)	139 (26.2)	21 (36.2)	0.105
ANC <1.5 × 10⁹/L	22 (3.7)	16 (3.0)	6 (10.3)	0.015
PLT <200 × 10⁹/L	276 (46.9)	239 (45.1)	37 (63.8)	0.007
Albumin <35 g/L	88 (15.0)	74 (14.0)	14 (24.1)	0.039

Values are median (IQR) or *n* (%). *P*-values were calculated using Mann–Whitney *U*, *χ*^2^, or Fisher exact tests, as appropriate. BMI was available for 585 patients (528 without and 57 with hematological readmission); BMI summaries use available cases. ANC, absolute neutrophil count; BMI, body mass index; IQR, interquartile range; PLT, platelet count.

### Treatment and supportive care characteristics

Before index discharge, 578 out of 588 patients (98.3%) had received chemotherapy, 540 (91.8%) had received induction chemotherapy, and 415 (70.6%) had received concurrent chemotherapy. The median cumulative cycle count was 5 (IQR 3–6); among chemotherapy-exposed patients, the median interval from the most recent chemotherapy administration to discharge was 20 days (IQR 7–52). Only one patient received adjuvant chemotherapy before index discharge. Detailed sequences, regimens, platinum, outcomes, and center distributions are shown in Supplementary Tables S2b and S16.

Among 520 patients with evaluable supportive care orders, G-CSF was recorded in 259 (49.8%), systemic antimicrobial treatment in 98 (18.8%), and nutritional support in 105 (20.2%). Red-cell and platelet transfusions were each recorded in one patient; human albumin infusion was recorded in 6 out of 518 evaluable patients. Sixty-eight records from Center 3 had structurally unavailable order histories and remained missing.

### Core risk marker burden

Hematological toxicity-related readmission occurred in 13 out of 263 patients (4.9%) with no core markers, 35 out of 268 (13.1%) with one marker, and 10 out of 57 (17.5%) with two or more markers (Figure 2). In the primary adjusted Firth model, ORs were 2.86 (95% CI 1.50–5.46; *P* = 0.001) and 4.33 (1.82–10.30; *P* < 0.001) for one and two or more versus zero markers, respectively (Table 2).

**Figure 2:**
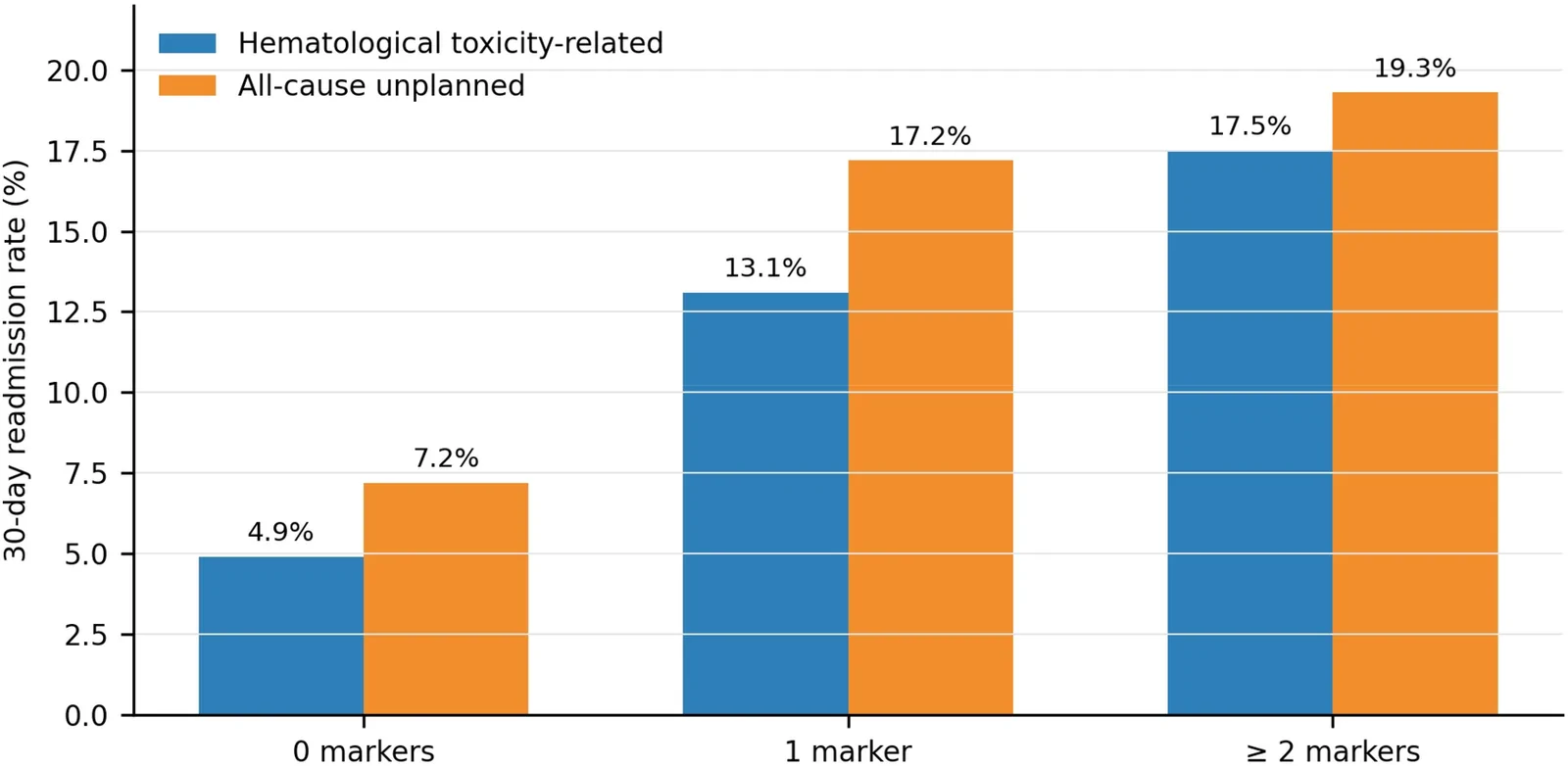
Observed hematological toxicity-related and all-cause unplanned readmission rates by number of core predischarge risk markers in the primary analytic sample (*N* = 588). Core markers were ANC < 1.5 × 10⁹/L, PLT < 200 × 10⁹/L, and albumin <35 g/L. ANC, absolute neutrophil count; PLT, platelet count.

**Table 2:** Core risk marker burden and adjusted Firth estimates for the primary and supportive secondary outcomes.

Risk marker stratum	*N*	Hematological toxicity-related readmission, *n* (%)	Adjusted OR (95% CI)	All-cause unplanned readmission, *n* (%)	Adjusted OR (95% CI)
0 markers	263	13 (4.9)	Reference	19 (7.2)	Reference
1 marker	268	35 (13.1)	2.86 (1.50–5.46); *P* = 0.001	46 (17.2)	2.65 (1.52–4.61); *P* < 0.001
≥2 markers	57	10 (17.5)	4.33 (1.82–10.30); *P* < 0.001	11 (19.3)	3.18 (1.43–7.05); *P* = 0.004

Both outcomes used the clinical comparator adjustment set: age, sex, stage, missing-stage indicator, and comorbidity. The all-cause analysis included 76 events in the 588-patient sample and was a supportive same-cohort analysis, not external validation. CI, confidence interval; OR, odds ratio.

### Association rule mining and Firth regression

Eleven rules met the screening thresholds before the redundancy review. The retained combinations were comorbidity plus albumin < 35 g/L (antecedent *n* = 48; events *n* = 10; confidence 20.8%; lift 2.11; adjusted OR 2.75, 95% CI 1.29–5.84), Hb < 100 g/L plus albumin < 35 g/L (*n* = 60; events *n* = 11; confidence 18.3%; lift 1.86; OR 2.41, 95% CI 1.18–4.96), and Hb < 100 g/L plus PLT < 200 × 10⁹/L (*n* = 76; events *n* = 12; confidence 15.8%; lift 1.60; OR 1.98, 95% CI 1.00–3.91). The third rule was considered exploratory (Table 3; Figure 3).

**Table 3:** Retained association rules and adjusted Firth logistic regression estimates.

Rule	Antecedent	*N*	Events	Support, %	Confidence, %	Lift	Adjusted OR (95% CI)
R1	Comorbidity+albumin <35 g/L	48	10	1.7	20.8	2.11	2.75 (1.29–5.84); *P* = 0.009
R2	Hb < 100 g/L + albumin <35 g/L	60	11	1.9	18.3	1.86	2.41 (1.18–4.96); *P* = 0.016
R3	Hb < 100 g/L + PLT <200 × 10⁹/L	76	12	2.0	15.8	1.60	1.98 (1.00–3.91); *P* = 0.050

The analysis included *N* = 588 with 58 primary events. Rules used support ≥1.5%, confidence ≥15%, lift ≥1.5, antecedent length ≤3, and ≥9 events. ORs were adjusted for the clinical comparator covariates unless a covariate was included in the rule antecedent. CI, confidence interval; Hb, hemoglobin; OR, odds ratio; PLT, platelet count.

**Figure 3:**
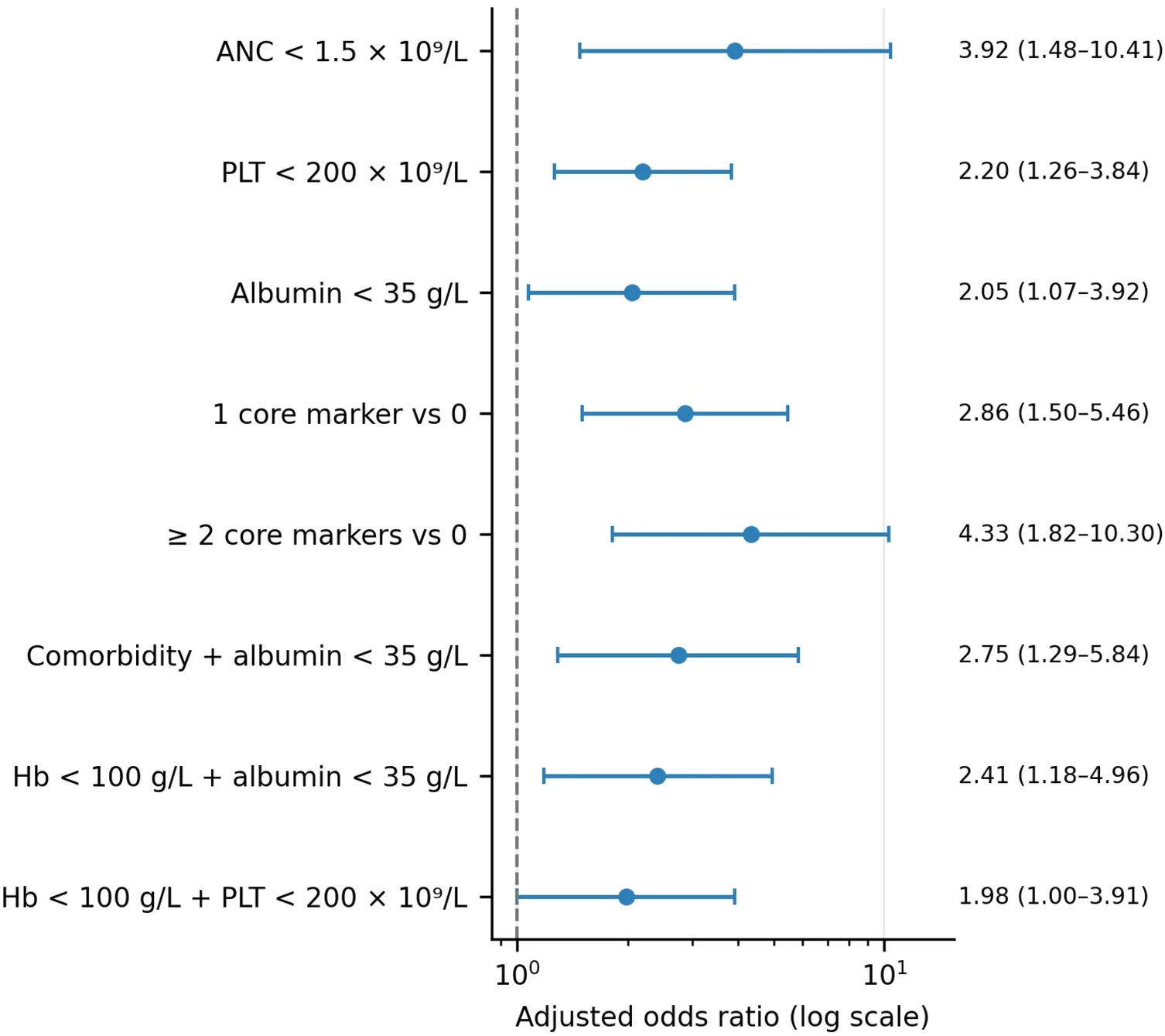
Adjusted associations of selected indicators, risk marker strata, and retained rules with hematological toxicity-related readmission in the primary analytic sample (*N* = 588; 58 events). Points show adjusted ORs, horizontal bars show 95% CIs, and the dashed vertical line denotes OR=1. Estimates used the clinical comparator adjustment set unless a covariate was part of a rule antecedent. ANC, absolute neutrophil count; CI, confidence interval; Hb, hemoglobin; OR, odds ratio; PLT, platelet count.

### Treatment, supportive care, and threshold sensitivity analyses

Additional treatment adjustment did not materially change the marker burden gradient. In Model C1 (*N* = 588; 58 events), adjusted ORs were 2.93 (95% CI 1.52–5.64) and 4.55 (1.88–11.02); cumulative cycle count and concurrent chemotherapy were not clearly associated with the outcome. In Model C2 among chemotherapy recipients (*N* = 578; 57 events), corresponding ORs were 2.84 (1.47–5.49) and 4.51 (1.85–11.03); the cycle count and interval from chemotherapy to discharge were not clearly associated. Concurrent-chemotherapy strata showed the same direction but had wide confidence intervals (Supplementary Table S17).

In the supportive care-evaluable subset (*N* = 520; 57 events), S0 ORs were 2.64 (95% CI 1.37–5.07) and 4.27 (1.77–10.25). After adding G-CSF, antimicrobial treatment, and nutritional support (S1), ORs were 2.59 (1.34–5.02) and 3.47 (1.38–8.76). G-CSF and antimicrobial coefficients were near the null; nutritional support was associated with higher odds (OR 2.41, 1.33–4.38), a finding treated as confounding by indication rather than a causal adverse effect.

With PLT < 150 × 10⁹/L, the adjusted OR was 1.71 (95% CI 0.93–3.13; *P* = 0.081) for one marker and 3.40 (1.45–7.98; *P* = 0.005) for two or more. In separate continuous models, ORs per lower value were 0.97 (0.93–1.02) per 1 × 10⁹/L ANC, 1.16 (0.96–1.41) per 50 × 10⁹/L PLT, and 1.32 (0.95–1.84) per 5 g/L albumin; all three 95% CIs included 1. These results do not identify an optimal laboratory threshold (Supplementary Table S18).

### Internal model performance and all-cause readmission

The clinical comparator had an apparent AUC of 0.537. Adding all three binary core markers yielded an apparent AUC of 0.673; the marker burden and rule count models yielded 0.659 and 0.638, respectively. Bootstrap refitting reduced performance estimates, resulting in an optimism-corrected AUC of 0.615 for marker burden. Corrected calibration slopes were below 1, indicating overfitting and reinforcing the need for independent validation (Table 4).

**Table 4:** Apparent and bootstrap optimism-corrected internal performance of clinical and marker-derived models.

Model	Apparent AUC (95% CI)	Optimism-corrected AUC	Apparent *Δ*AUC vs. comparator (95% CI)	Brier score, apparent/corrected	Calibration slope, apparent/corrected
Clinical comparator	0.537 (0.454–0.614)	0.476	Reference	0.0886/0.0903	0.888/0.166
Comparator+continuous ANC	0.562 (0.477–0.641)	0.495	0.025 (−0.022–0.071)	0.0885/0.0906	0.892/0.228
Comparator+continuous PLT	0.619 (0.549–0.694)	0.557	0.082 (0.013–0.156)	0.0882/0.0903	0.971/0.393
Comparator+continuous albumin	0.582 (0.507–0.661)	0.522	0.045 (−0.029–0.122)	0.0883/0.0904	0.959/0.432
Comparator+all three continuous measures	0.626 (0.558–0.703)	0.555	0.090 (0.005–0.181)	0.0879/0.0909	0.977/0.468
Comparator+all three binary core markers	0.673 (0.608–0.734)	0.624	0.136 (0.042–0.230)	0.0864/0.0898	0.993/0.717
Comparator+marker burden strata	0.659 (0.596–0.720)	0.615	0.122 (0.029–0.211)	0.0869/0.0896	1.004/0.732
Comparator+rule count strata	0.638 (0.566–0.708)	0.585	0.102 (0.020–0.190)	0.0870/0.0899	0.982/0.674

All models used *N* = 588 with 58 primary events. The clinical comparator comprised age, sex, stage, the missing-stage indicator, and comorbidity. Probability metrics used FLIC. Calibration-in-the-large was 0.000, and the mean predicted risk equaled the observed risk (0.099) for each apparent FLIC model. Calibration-in-the-large was calculated with the calibration slope fixed at 1; the separate calibration intercepts reported in Supplementary Table S19 were estimated jointly with the calibration slope. Optimism correction involved repeated coefficient fitting in 1,000 bootstrap samples. Rule discovery and pruning were not repeated, so the rule count estimate remains conditional on the retained rules. AUC, area under the receiver operating characteristic curve; CI, confidence interval; FLIC, Firth's logistic regression with intercept correction; *Δ*, difference.

Exploratory decision curve analysis showed an apparent net benefit for the marker burden model over the clinical comparator and treat-all and treat-none strategies across threshold probabilities of approximately 0.04–0.13. This development-sample finding was not interpreted as demonstrating clinical utility. Apparent calibration and decision curves are shown in Supplementary Figures S2 and S3.

In the supportive secondary analysis, all-cause unplanned readmission occurred in 19/263 patients (7.2%) with no marker, 46/268 (17.2%) with one marker, and 11/57 (19.3%) with two or more markers. Adjusted ORs were 2.65 (95% CI 1.52–4.61) and 3.18 (95% CI 1.43–7.05), respectively (Table 2; Figure 2). Because the all-cause outcome included all primary events in the same cohort, this analysis reduced reliance on cause classification but did not provide independent validation. Cumulative hematological readmission proportions over 30 days are shown in Figure 4.

**Figure 4:**
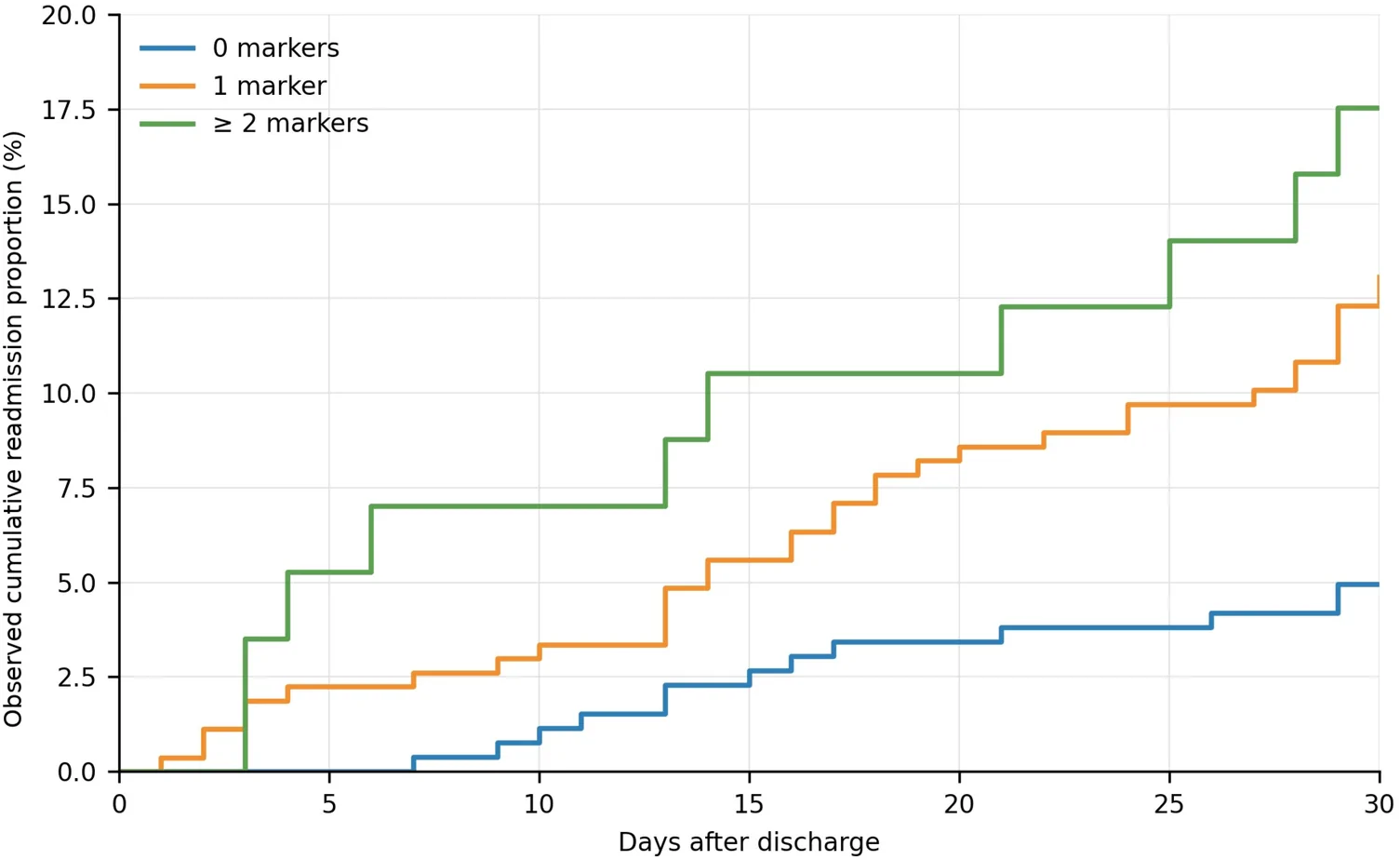
Observed cumulative hematological toxicity-related readmission by core marker burden over 30 days in the primary analytic sample (*N* = 588). Core markers were ANC <1.5 × 10⁹/L, PLT <200 × 10⁹/L, and albumin <35 g/L. Follow-up was administratively censored at 30 days. ANC, absolute neutrophil count; PLT, platelet count.

## Discussion

In this multicenter cohort, approximately three-quarters of recorded 30-day unplanned readmissions were classified as hematological toxicity-related. A three-item marker burden identified groups with observed hematological readmission rates of approximately 5%, 13%, and 18%. Because predischarge blood count abnormalities may represent the same unresolved toxicity process that later prompts admission, these associations should not be interpreted as causal or as evidence that events were preventable. The similar all-cause gradient supports broader prognostic relevance but does not constitute external validation or eliminate conceptual overlap.

Previous oncology studies have described rates, causes, and predictors of early readmission or potentially avoidable hospitalization (5–12). The 12.7% all-cause unplanned readmission rate in our full cohort was close to the 13.8% reported after head and neck cancer hospitalization (6) and lower than the 22.6% reported in a general medical oncology service (8), illustrating the dependence of readmission rates on case mix and care setting. More broadly, retrospective oncology research has examined the prognostic value of baseline laboratory parameters for survival. In advanced cancer, Deme et al. found that C-reactive protein, albumin, and platelet-to-lymphocyte ratio, particularly when considered in combination, were associated with overall survival (42); although survival and early readmission are distinct outcomes, this work provides broader context for the concept that routine laboratory measures may jointly reflect clinically relevant patient vulnerability. Unlike these broader studies, we focused on a cause-specific endpoint and joint discharge-time marker patterns in patients with NPC. We evaluated transparent, internally assessed groupings of routine measures rather than proposing novel biomarkers. Low albumin appeared in higher-rate combinations with comorbidity or anemia. Albumin is non-specific and may reflect inflammation, illness burden, mucosal intake limitation, hydration, or treatment-related catabolism; it should not be treated as a stand-alone nutritional diagnosis (23–26, 30). Hb < 100 g/L also appeared in combinations with low albumin or PLT < 200 × 10⁹/L, suggesting pattern-level vulnerability rather than a single-marker mechanism.

Treatment and supportive care analyses addressed important measured confounding while preserving the prespecified primary model. The marker burden gradient was materially similar after adjustment for concurrent chemotherapy, cumulative cycles, and the chemotherapy-to-discharge interval. Supportive care adjustment attenuated the ≥ 2-marker estimate but did not eliminate the gradient. These analyses do not estimate treatment effects. In particular, G-CSF, antimicrobial treatment, and nutritional support may be initiated in response to clinical deterioration reflected by the same predischarge measurements; the nutritional-support association is therefore consistent with confounding by indication and temporal ambiguity.

The expanded performance comparison showed that the proposed models added apparent discrimination beyond the basic clinical comparator, which was not a validated clinical standard, but optimism correction reduced the AUC estimates and calibration slopes. Decision curves likewise reflect only the development sample. These results support further study of parsimonious marker groupings, rather than individual-level prediction or current clinical deployment. The rule count bootstrap refitted coefficients but did not repeat rule mining, so its performance may be more optimistic than reported.

Categorization was retained for interpretability but loses information and makes estimates dependent on the selected boundaries (32). PLT < 200 × 10⁹/L must not be interpreted as thrombocytopenia or used as a treatment threshold. With PLT < 150 × 10⁹/L, the association remained strongest for two or more markers, whereas the individual associations in continuous models were imprecise. The continuous ANC estimate also differed in direction from the binary low-ANC estimate; the small low-ANC subgroup and possible non-linearity further argue against interpreting either boundary as a clinical cutoff. PLT < 200 × 10⁹/L remains a prespecified exploratory marker and requires replication.

Several limitations should be acknowledged. First, retrospective routinely collected data are subject to residual confounding, misclassification, and site-specific practices. Second, core marker missingness was concentrated at Center 2 (18/35 records). Third, readmissions were captured only in the three participating hospitals; admissions elsewhere and deaths without a participating hospital admission may have been missed. Emergency visits without admission and outpatient transfusions were outside the endpoint. Fourth, although two researchers independently reviewed readmission classifications and reconciled disagreements by consensus, their initial individual ratings were not retained, precluding the calculation of inter-rater agreement or Cohen's *κ*. Fifth, 68 early Center 3 records lacked accessible supportive care orders, and this missingness was structural. Sixth, chemotherapy was nearly universal; regimen categories were sparse and center-dependent, cumulative drug dose was unavailable, and treatment first administered after discharge was not modeled as a time-varying exposure; treatment effects therefore cannot be separated from indication. Seventh, center sizes and event rates were imbalanced, with no primary event occurring in one center. Eighth, bootstrap optimism correction indicated overfitting, and no external validation was available. Ninth, all centers were tertiary hospitals in southwestern China, a high-incidence region for NPC; case mix, regimens, discharge practices, follow-up access, and readmission thresholds may differ across institutions, cancer types, regions, and health systems.

Future studies should prespecify the outcome dictionary, retain the reviewers’ individual ratings, and capture readmissions across health systems. They should also prospectively evaluate whether enhanced discharge transition support, including earlier laboratory reassessment, symptom calls, nutrition reviews, and rapid-access pathways, improves patient-centered and safety outcomes. Figure 5 summarizes a hypothesis-generating framework for such evaluation. Prospective studies should externally evaluate calibration and net benefit and should not assume that the current marker categories define treatment thresholds.

**Figure 5:**
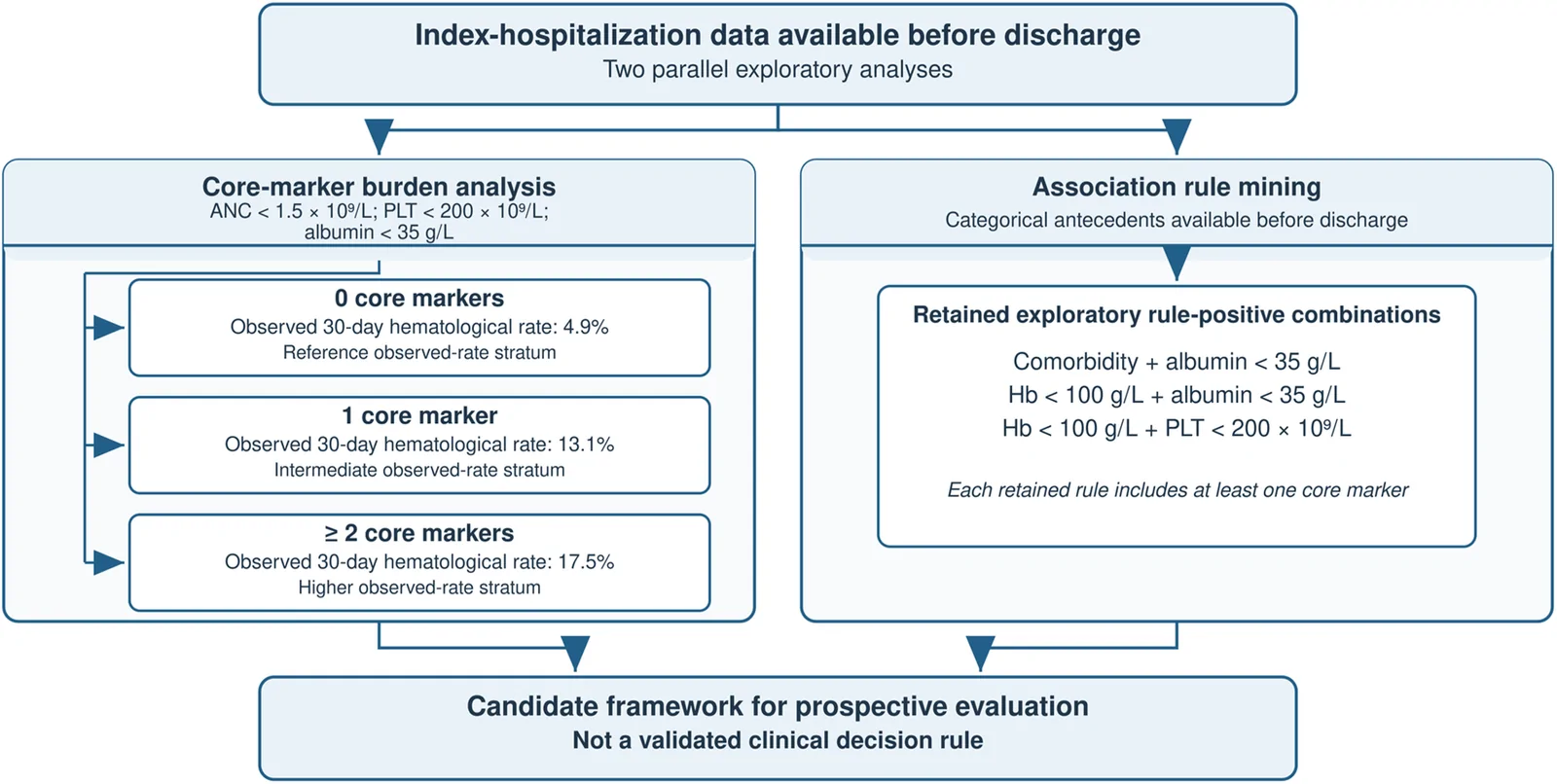
Hypothesis-generating framework for the prospective evaluation of discharge transition support based on two parallel analyses of predischarge data: core marker burden and association rule mining. The branches represent parallel analyses rather than sequential patient classification. Core markers were ANC <1.5 × 10⁹/L, PLT <200 × 10⁹/L, and albumin <35 g/L. Because every retained rule included either albumin <35 g/L or PLT < 200 × 10⁹/L, patients with zero core markers could not satisfy a retained rule. Laboratory indicators were measured during the 7-calendar-day window ending on and including index discharge. The displayed percentages are observed 30-day hematological toxicity-related unplanned readmission rates; the group labels denote observed-rate strata, not validated risk strata. The framework has not been externally validated and is not intended to direct current discharge decisions. ANC, absolute neutrophil count; Hb, hemoglobin; PLT, platelet count.

## Conclusion

In this retrospective multicenter cohort, routine predischarge hematological and albumin measures formed interpretable patterns associated with 30-day hematological toxicity-related unplanned readmission, with a similar gradient for all-cause unplanned readmission. These associations are exploratory and do not establish causality, preventability, or a clinical decision rule. They justify prospective external evaluation of discharge transition strategies but should not direct current clinical care without validation.

## Data Availability

The datasets analyzed are not publicly available because they contain patient-level clinical information from participating hospitals. De-identified data may be made available from the corresponding author on reasonable request and subject to institutional approval.
