# Supplementary material for: Predischarge blood count and nutritional risk marker patterns associated with 30-day unplanned readmissions for hematological toxicity after radiotherapy for nasopharyngeal carcinoma: a multicenter cohort study
Source: Front Med (Lausanne). 2026 Oct 14;13:1929774. doi: 10.3389/fmed.2026.1929774 (PMC13713644; doi:10.3389/fmed.2026.1929774)
Supplement: Supplementary file 1 [file Supplementaryfile1.docx]

Supplementary Material

# Abbreviations

ANC, absolute neutrophil count; AUC, area under the receiver operating characteristic curve; BMI, body mass index; CI, confidence interval; DCA, decision-curve analysis; FLIC, Firth's logistic regression with intercept correction; G-CSF, granulocyte colony-stimulating factor; Hb, hemoglobin; IQR, interquartile range; OR, odds ratio; PLT, platelet count; VIF, variance inflation factor.

# Supplementary Methods

**Software environment.** Primary modeling and internal performance assessment used R 4.4.2 (R Foundation for Statistical Computing, Vienna, Austria) with tidyverse 2.0.0, arules 1.7-8, logistf 1.26.0, pROC 1.18.5, and boot 1.3-31. Data harmonization, sensitivity analyses, and independent checks used Python 3.11.9 with pandas 2.2.3, NumPy 1.26.4, SciPy 1.13.1, scikit-learn, Matplotlib 3.9.2, and a documented adjusted-score implementation of Firth regression. The resampling seed was 20251028.

**Data structure and preprocessing.** The unit of analysis was the first eligible index hospitalization, and each patient contributed one record. The primary complete-case sample required ANC, PLT, and albumin values during the 7-calendar-day window ending on and including index discharge. The measurement closest to discharge was selected. Blood cell counts were expressed as × 10⁹/L and albumin and hemoglobin as g/L. Core markers were not imputed; missing stage was represented by a separate indicator.

**Outcome and predictor coding.** Hematological toxicity-related unplanned readmission within 30 days was coded as 1; absence of a qualifying event was coded as 0. For patients with more than one readmission within 30 days, only the chronologically first readmission was used to determine patient-level outcome status. If the first readmission was for planned treatment continuation, it was coded as a non-event, and subsequent readmissions did not redefine the outcome. If the first readmission was unplanned, its principal documented reason determined the mutually exclusive readmission category; no severity-based or cause-priority hierarchy was applied. Binary predictors were coded 1 for presence and 0 for absence. The core markers were ANC < 1.5 × 10⁹/L, PLT < 200 × 10⁹/L, and albumin < 35 g/L; PLT < 150 × 10⁹/L was the conventional-threshold sensitivity definition. Predictor values were not used to assign the readmission-reason category.

**Chemotherapy and supportive care.** Any chemotherapy required a documented administration before index discharge. Induction, concurrent, and pre-discharge adjuvant chemotherapy were defined by timing relative to radiotherapy. Internally discordant summary fields were reconciled against dated administrations and documented refusal. Supportive-care exposures were assessed during the same 7-calendar-day window ending on and including index discharge to characterize care proximal to discharge. For each exposure, a documented administration was coded 1, no administration in an accessible executed-order history was coded 0, and an inaccessible history was coded missing.

**Descriptive comparisons.** Continuous variables were summarized as medians and IQRs and compared with Mann–Whitney U tests. Categorical variables were summarized as n (%) and compared with Pearson χ² or Fisher exact tests as appropriate. Tests were two-sided with P < 0.05; multiplicity adjustment was not applied because the analyses were exploratory.

**Association rule mining.** The Apriori algorithm was applied with hematological toxicity-related readmission fixed as the consequent. Screening required co-occurrence support ≥ 1.5%, confidence ≥ 15%, lift ≥ 1.5, antecedent length 1–3, and at least nine primary events. Antecedent support was the proportion satisfying the antecedent; leverage was the observed co-occurrence support minus the product of antecedent and outcome supports; conviction was (1 − outcome support)/(1 − confidence). Redundant longer rules were pruned in favor of simpler rules. Unadjusted Pearson χ² P values in the complete output were descriptive and were not retention criteria.

**Firth regression and internal performance.** Association estimates used Firth penalized logistic regression with profile penalized-likelihood 95% CIs and penalized likelihood-ratio P values. The basic clinical comparator comprised age per 10 years, sex, stage, missing stage, and comorbidity and was not presented as a validated standard clinical model. FLIC probabilities were used for Brier score, calibration, and DCA. Apparent AUC 95% CIs, paired AUC differences, and optimism correction used 1,000 patient-level bootstrap resamples with model refitting. Rule discovery and pruning were not repeated, so rule-count performance remains conditional on the retained rules.

**Sensitivity analyses.** Analyses excluded planned treatment-continuation admissions; used PLT < 150 × 10⁹/L; modeled laboratory measures continuously; restricted the sample to patients with complete stage data; adjusted for center; excluded the zero-event center; incorporated chemotherapy or supportive-care variables; and used all-cause 30-day unplanned readmission as a supportive secondary outcome. DCA evaluated thresholds from 0.03 to 0.30 and was exploratory.

# Supplementary Figures

**Supplementary Figure S1.** Apparent discrimination of selected comparator and marker-grouping models for hematological toxicity-related unplanned readmission (N = 588; 58 events). Points show AUC estimates; horizontal bars show 95% confidence intervals. The dashed line denotes an AUC of 0.5. In the plot, ‘All 3 binary markers,’ ‘Marker burden,’ and ‘Rule count’ denote the basic clinical comparator plus the corresponding marker representation. AUC, area under the receiver operating characteristic curve.

**Supplementary Figure S2.** Apparent calibration of selected FLIC probability models for hematological toxicity-related unplanned readmission (N = 588; 58 events): (A) clinical comparator; (B) comparator plus all three binary core markers; (C) comparator plus marker-burden strata; and (D) comparator plus rule-count strata. Points and connecting lines show mean predicted probability and observed event proportion within predicted-risk quintiles; dashed lines denote ideal calibration. These are development-sample summaries; bootstrap-corrected slopes are reported in Supplementary Table S19. FLIC, Firth's logistic regression with intercept correction.

**Supplementary Figure S3.** Exploratory decision-curve analysis for hematological toxicity-related unplanned readmission (N = 588; 58 events). Apparent net benefit is shown for the basic clinical comparator and models adding all three binary core markers, marker-burden strata, or rule-count strata across threshold probabilities from 0.03 to 0.30. These development-sample curves do not establish clinical utility or implementation readiness.

# Supplementary Tables

**Supplementary Table S1.** Prespecified analytic plan.

| **Component** | **Prespecified specification** | **Rationale/implementation** |
| --- | --- | --- |
| Primary analytic sample | Complete ANC, platelet count, and albumin measured within the 7-calendar-day window ending on and including index discharge | N = 588; for patients with multiple measurements during the 7-calendar-day window ending on and including index discharge, the value closest to discharge was selected |
| Primary outcome | Hematological toxicity-related 30-day unplanned readmission | Consensus readmission-reason category: hematological toxicity/myelosuppression |
| Secondary outcome | All-cause 30-day unplanned readmission | Documented unplanned readmission within 30 days after discharge |
| Planned admissions | Not counted as unplanned events | Sensitivity analysis excludes planned treatment continuation admissions |
| Core risk-marker thresholds | ANC < 1.5 × 10⁹/L; PLT < 200 × 10⁹/L; Albumin < 35 g/L | PLT < 200 × 10⁹/L was treated as a prespecified exploratory marker; PLT < 150 × 10⁹/L was used as a sensitivity threshold |
| Association rule support | ≥ 1.5% co-occurrence support | Corresponding to ≥ 9 patients with both the antecedent and outcome in the primary analytic sample (N = 588). |
| Association rule confidence | ≥ 15% | Above baseline hematological readmission risk of 9.9% |
| Association rule lift | ≥ 1.5 | At least 1.5-fold higher than baseline risk |
| Rule length | Antecedent length 1–3 items | Main interpretation prioritizes parsimonious 2-item rules |
| Minimum event count | ≥ 9 hematological readmission events per main rule | Rules below this threshold were not retained as qualifying rules |
| Evaluation | Firth penalized logistic regression | Adjusted for age per 10 years, sex, stage, missing-stage indicator, and comorbidity unless included in rule |
| Internal model evaluation | Apparent and optimism-corrected AUC, Brier score, calibration, and exploratory decision-curve analysis | Exploratory internal performance assessment only; no external validation |
| Data harmonization | Center-level variable mapping and unit harmonization using a common data dictionary | Variables were mapped centrally; laboratory units were harmonized across centers; laboratory/BMI/threshold variables were selected from the most recent measurement within the 7-calendar-day window ending on and including index discharge; no z-score standardization was used because absolute prespecified cut-points and marker definitions were the target variables |
| Statistical software | R 4.4.2 and Python 3.11.9 | Primary modeling and internal performance assessment used R; data harmonization, sensitivity analyses, and independent verification used Python. |
| Sparse-data/model diagnostics | Sparse-data and collinearity safeguards | Firth logistic regression was used to reduce small-sample/separation bias; collinearity was addressed by domain-based variable selection, dummy coding with reference categories, avoidance of redundant laboratory markers, and sensitivity models to check stability. |

**Note:** The association-rule thresholds, complete-case primary sample, and core markers were prespecified before the sensitivity analyses.

**Supplementary Table S2.** Sample flow and analytic samples.

| **Item** | **N** | **Note** |
| --- | --- | --- |
| Included full cohort with classifiable 30-day readmission status in participating-hospital records | 607 | Full cohort |
| Patients with ANC, platelet count, and albumin complete | 588 | Primary analytic sample |
| Patients excluded because one or more core variables were missing | 19 | 18 albumin-only missing; 1 all three missing |
| 30-day unplanned readmissions in the full cohort | 77 | 12.7% |
| 30-day hematological toxicity-related unplanned readmissions in the full cohort | 58 | 9.6% of the full cohort; 58/77 = 75.3% of unplanned readmissions |
| Planned treatment continuation admissions in the full cohort | 38 | Treated as non-events in primary analysis; excluded in sensitivity analysis |
| 30-day unplanned readmissions in the primary analytic sample | 76 | 12.9% |
| Hematological toxicity-related unplanned readmissions in the primary analytic sample | 58 | 9.9% |

**Note:** Planned treatment-continuation admissions were non-events. Readmissions were captured from the electronic medical record systems of the three participating hospitals. The one all-cause unplanned readmission outside the primary sample occurred in a patient with missing core-marker data and was not classified as hematological toxicity-related.

**Supplementary Table S2a.** Center-level cohort, outcomes, and core-marker missingness.

| **Center** | **Full cohort N** | **Primary sample N** | **Missing core markers n (%)** | **Hematological readmission n (%)** | **All-cause unplanned n (%)** |
| --- | --- | --- | --- | --- | --- |
| Center 1 | 432 | 431 | 1 (0.2) | 49 (11.3) | 59 (13.7) |
| Center 2 | 35 | 17 | 18 (51.4) | 0 (0.0) | 2 (5.7) |
| Center 3 | 140 | 140 | 0 (0.0) | 9 (6.4) | 16 (11.4) |
| Total | 607 | 588 | 19 (3.1) | 58 (9.6) | 77 (12.7) |

**Note:** Readmission percentages use the full center cohort. Within the 607-patient included cohort, 30-day readmission-status missingness under the participating-hospital record definition was 0 at each center. Readmissions to non-participating hospitals and deaths without a participating-hospital admission could not be captured.

**Supplementary Table S2b.** Center-level chemotherapy and supportive-care patterns in the primary sample.

| **Center** | **N** | **Any chemotherapy n/N (%)** | **Induction n (%)** | **Concurrent n (%)** | **Cycles median (IQR)** | **G-CSF n/evaluable N (%)** | **Antimicrobial n/evaluable N (%)** | **Nutritional support n/evaluable N (%)** | **Structurally unavailable order histories, n** |
| --- | --- | --- | --- | --- | --- | --- | --- | --- | --- |
| Center 1 | 431 | 423/431 (98.1) | 394 (91.4) | 285 (66.1) | 5.0 (3.0–7.0) | 227/431 (52.7) | 91/431 (21.1) | 93/431 (21.6) | 0 |
| Center 2 | 17 | 16/17 (94.1) | 14 (82.4) | 6 (35.3) | 3.0 (2.0–4.0) | 2/17 (11.8) | 2/17 (11.8) | 2/17 (11.8) | 0 |
| Center 3 | 140 | 139/140 (99.3) | 132 (94.3) | 124 (88.6) | 4.5 (3.8–5.0) | 30/72 (41.7) | 5/72 (6.9) | 10/72 (13.9) | 68 |

**Note:** Supportive-care columns report n/evaluable N (%) for recorded exposure. Center 3 had 68 early records with structurally unavailable order histories. Human albumin infusion data were additionally missing for two Center 1 patients. Regimen categories were descriptive because they were sparse and strongly center-linked. One pre-discharge adjuvant-chemotherapy exposure occurred at Center 1.

**Supplementary Table S2c.** Conceptual separation of candidate predictors and outcome definitions.

| **Component** | **Source field or time window** | **Role in analysis** | **Circularity safeguard** |
| --- | --- | --- | --- |
| Candidate pre-discharge markers | Index hospitalization; most recent laboratory or anthropometric measurement within the 7-calendar-day window ending on and including index discharge | Antecedents/exposures in association rules and Firth models | Not used to classify the readmission reason or define hematological toxicity-related readmission |
| Primary outcome | Post-discharge readmission status, readmission date, and cleaned electronic medical record readmission-reason category within 30 days after discharge | Consequent/outcome: hematological toxicity-related unplanned readmission | Defined from readmission-reason categories, not from the pre-discharge predictor values |
| Planned treatment-continuation admissions | Electronic medical-record readmission-reason category | Not counted as unplanned events in the primary analysis; excluded in sensitivity analysis | Separated from acute unplanned readmission categories |
| All-cause unplanned readmission sensitivity outcome | Post-discharge unplanned readmission indicator within 30 days after discharge | Assesses whether the risk-marker gradient persists with an outcome that does not depend on hematological cause classification | Does not use the hematological cause category. |

**Note:** The primary outcome was a post-discharge hospital-utilization event. Pre-discharge values were used only as risk markers and were not used to assign the readmission-reason category.

**Supplementary Table S3.** Thirty-day readmission status in the full cohort.

| **Mutually exclusive status** | **N** | **Percent of full cohort, %** | **Analysis status** |
| --- | --- | --- | --- |
| Unplanned readmission | 77 | 12.7 | All-cause outcome |
| Planned treatment-continuation admission | 38 | 6.3 | Non-event |
| No recorded 30-day readmission | 492 | 81.1 | Non-event |
| Full cohort | 607 | 100.0 | Total |

**Note:** Categories are mutually exclusive and use the full cohort denominator (N = 607). Percentages may not sum to 100.0% because of rounding. Readmissions to non-participating hospitals were not captured.

**Supplementary Table S4.** Baseline characteristics of the primary analytic sample.

| **Variable** | **Overall (N = 588)** | **No hematological readmission (N = 530)** | **Hematological readmission (N = 58)** | **P value** | **Summary type** |
| --- | --- | --- | --- | --- | --- |
| Age, years | 50.0 (43.0, 57.0) | 50.0 (43.0, 57.0) | 49.0 (42.2, 54.0) | 0.461 | Median (IQR) |
| BMI, kg/m² | 23.1 (20.9, 25.5) | 23.2 (20.9, 25.5) | 22.4 (20.5, 24.9) | 0.210 | Median (IQR) |
| ANC, × 10⁹/L | 3.74 (2.70, 5.96) | 3.73 (2.71, 5.79) | 4.00 (2.37, 7.68) | 0.838 | Median (IQR) |
| PLT, × 10⁹/L | 206.0 (160.0, 261.2) | 208.5 (161.0, 265.0) | 185.0 (155.2, 231.5) | 0.024 | Median (IQR) |
| Albumin, g/L | 39.1 (36.5, 41.7) | 39.1 (36.7, 41.8) | 39.2 (35.4, 41.4) | 0.294 | Median (IQR) |
| Hemoglobin, g/L | 111.0 (98.0, 122.0) | 111.0 (99.0, 122.0) | 108.5 (91.5, 118.0) | 0.078 | Median (IQR) |
| Lymphocyte count, × 10⁹/L | 0.4 (0.2, 0.5) | 0.4 (0.2, 0.5) | 0.4 (0.2, 0.5) | 0.797 | Median (IQR) |
| Length of stay, days | 46.0 (40.0, 51.0) | 46.0 (40.0, 51.0) | 46.5 (39.8, 53.0) | 0.242 | Median (IQR) |
| Male sex | 427 (72.6) | 388 (73.2) | 39 (67.2) | 0.333 | n (%) |
| Age ≥ 60 years | 94 (16.0) | 88 (16.6) | 6 (10.3) | 0.217 | n (%) |
| Advanced clinical stage (III–IV) | 530 (90.1) | 479 (90.4) | 51 (87.9) | 0.553 | n (%) |
| Missing clinical stage | 10 (1.7) | 9 (1.7) | 1 (1.7) | 1.000 | n (%) |
| Comorbidity | 280 (47.6) | 252 (47.5) | 28 (48.3) | 0.916 | n (%) |
| ANC < 1.5 × 10⁹/L | 22 (3.7) | 16 (3.0) | 6 (10.3) | 0.015 | n (%) |
| PLT < 200 × 10⁹/L | 276 (46.9) | 239 (45.1) | 37 (63.8) | 0.007 | n (%) |
| Albumin < 35 g/L | 88 (15.0) | 74 (14.0) | 14 (24.1) | 0.039 | n (%) |
| PLT < 150 × 10⁹/L | 111 (18.9) | 97 (18.3) | 14 (24.1) | 0.281 | n (%) |
| Hemoglobin < 100 g/L | 160 (27.2) | 139 (26.2) | 21 (36.2) | 0.105 | n (%) |
| BMI < 18.5 kg/m² | 35 (6.0) | 29 (5.5) | 6 (10.5) | 0.138 | n (%) |

**Note:** Laboratory and anthropometric variables used the measurement closest to discharge during the 7-calendar-day window ending on and including index discharge. P values were calculated with Mann–Whitney U, χ², or Fisher exact tests, as appropriate. BMI was available for 585 patients and lymphocyte count for 587; these summaries used available cases. Missing stage was retained as a missingness indicator.

**Supplementary Table S5.** Threshold-specific hematological toxicity-related readmission rates.

| **Indicator** | **Positive N** | **Hematological events** | **Hematological readmission rate** | **95% CI lower** | **95% CI upper** | **Lift vs baseline** |
| --- | --- | --- | --- | --- | --- | --- |
| ANC < 1.5 × 10⁹/L | 22 | 6 | 0.273 | 0.132 | 0.482 | 2.76 |
| PLT < 200 × 10⁹/L | 276 | 37 | 0.134 | 0.099 | 0.179 | 1.36 |
| Albumin < 35 g/L | 88 | 14 | 0.159 | 0.097 | 0.250 | 1.61 |
| PLT < 150 × 10⁹/L | 111 | 14 | 0.126 | 0.077 | 0.201 | 1.28 |
| Hemoglobin < 100 g/L | 160 | 21 | 0.131 | 0.087 | 0.192 | 1.33 |
| BMI < 18.5 kg/m² | 35 | 6 | 0.171 | 0.081 | 0.327 | 1.74 |

**Note:** Threshold variables used the measurement closest to discharge during the 7-calendar-day window ending on and including index discharge. Confidence intervals are Wilson 95% confidence intervals.

**Supplementary Table S6.** Complete association-rule output with hematological toxicity-related readmission fixed as the consequent.

| **Antecedent** | **Length** | **Antecedent N** | **Event N** | **Support** | **Antecedent support** | **Confidence** | **Lift** | **Leverage** | **Conviction** | **P value** |
| --- | --- | --- | --- | --- | --- | --- | --- | --- | --- | --- |
| Advanced stage (III–IV) + Albumin < 35 g/L | 2 | 77 | 14 | 0.024 | 0.131 | 0.182 | 1.84 | 0.011 | 1.10 | 0.009 |
| Albumin < 35 g/L | 1 | 88 | 14 | 0.024 | 0.150 | 0.159 | 1.61 | 0.009 | 1.07 | 0.039 |
| Hemoglobin < 100 g/L + PLT < 200 × 10⁹/L | 2 | 76 | 12 | 0.020 | 0.129 | 0.158 | 1.60 | 0.008 | 1.07 | 0.063 |
| Advanced stage (III–IV) + Comorbidity + Hemoglobin < 100 g/L | 3 | 76 | 12 | 0.020 | 0.129 | 0.158 | 1.60 | 0.008 | 1.07 | 0.063 |
| Advanced stage (III–IV) + Hemoglobin < 100 g/L + Albumin < 35 g/L | 3 | 53 | 11 | 0.019 | 0.090 | 0.208 | 2.10 | 0.010 | 1.14 | 0.005 |
| Hemoglobin < 100 g/L + Albumin < 35 g/L | 2 | 60 | 11 | 0.019 | 0.102 | 0.183 | 1.86 | 0.009 | 1.10 | 0.020 |
| Advanced stage (III–IV) + Hemoglobin < 100 g/L + PLT < 200 × 10⁹/L | 3 | 66 | 11 | 0.019 | 0.112 | 0.167 | 1.69 | 0.008 | 1.08 | 0.049 |
| Advanced stage (III–IV) + Comorbidity + Albumin < 35 g/L | 3 | 40 | 10 | 0.017 | 0.068 | 0.250 | 2.53 | 0.010 | 1.20 | < 0.001 |
| Comorbidity + Albumin < 35 g/L | 2 | 48 | 10 | 0.017 | 0.082 | 0.208 | 2.11 | 0.009 | 1.14 | 0.008 |
| Male sex + Advanced stage (III–IV) + Albumin < 35 g/L | 3 | 54 | 9 | 0.015 | 0.092 | 0.167 | 1.69 | 0.006 | 1.08 | 0.079 |
| Male sex + Hemoglobin < 100 g/L + PLT < 200 × 10⁹/L | 3 | 55 | 9 | 0.015 | 0.094 | 0.164 | 1.66 | 0.006 | 1.08 | 0.090 |

**Note:** Rules were screened with co-occurrence support, confidence, lift, antecedent-length, and event-count requirements. Support, antecedent support, and confidence are proportions. Leverage is observed co-occurrence support minus the product of antecedent and outcome supports; conviction is (1 − outcome support)/(1 − confidence). P values are unadjusted Pearson χ² test results and are descriptive only.

**Supplementary Table S7.** Retained association-rule metrics.

| **Main rule** | **Antecedent N** | **Event N** | **Support** | **Confidence** | **Lift** | **Reason selected** |
| --- | --- | --- | --- | --- | --- | --- |
| R1: Comorbidity + Albumin < 35 g/L | 48 | 10 | 0.017 | 0.208 | 2.11 | Parsimonious clinically interpretable rule |
| R2: Hemoglobin < 100 g/L + Albumin < 35 g/L | 60 | 11 | 0.019 | 0.183 | 1.86 | Parsimonious clinically interpretable rule |
| R3: Hemoglobin < 100 g/L + PLT < 200 × 10⁹/L | 76 | 12 | 0.020 | 0.158 | 1.60 | Parsimonious clinically interpretable rule |

**Note:** Rules were retained for parsimony, interpretability, and event count. Support and confidence are proportions.

**Supplementary Table S8.** Firth logistic regression results for individual pre-discharge markers.

| **Model exposure** | **Term** | **OR** | **CI lower** | **CI upper** | **P value** | **N** | **Events** | **Converged** | **Iterations** |
| --- | --- | --- | --- | --- | --- | --- | --- | --- | --- |
| ANC < 1.5 × 10⁹/L | ANC_low_1_5 | 3.92 | 1.48 | 10.41 | 0.006 | 588 | 58 | Yes | 9 |
| PLT < 200 × 10⁹/L | PLT_low_200 | 2.20 | 1.26 | 3.84 | 0.005 | 588 | 58 | Yes | 9 |
| Albumin < 35 g/L | ALB_low_35 | 2.05 | 1.07 | 3.92 | 0.030 | 588 | 58 | Yes | 9 |
| Hemoglobin < 100 g/L | Hb_low_100 | 1.67 | 0.93 | 2.97 | 0.084 | 588 | 58 | Yes | 9 |
| PLT < 150 × 10⁹/L (sensitivity threshold) | PLT_low_150 | 1.49 | 0.79 | 2.80 | 0.215 | 588 | 58 | Yes | 9 |

**Note:** ORs were adjusted as specified in the manuscript. CI, confidence interval; OR, odds ratio.

**Supplementary Table S9.** Firth logistic regression results for core marker burden and rule-count strata.

| **Model exposure** | **Term** | **OR** | **CI lower** | **CI upper** | **P value** | **N** | **Events** | **Converged** | **Iterations** |
| --- | --- | --- | --- | --- | --- | --- | --- | --- | --- |
| Core risk-marker burden (ref: 0) | core_marker_1 | 2.86 | 1.50 | 5.46 | 0.001 | 588 | 58 | Yes | 10 |
| Core risk-marker burden (ref: 0) | core_marker_ge2 | 4.33 | 1.82 | 10.30 | < 0.001 | 588 | 58 | Yes | 10 |
| R1: Comorbidity + Albumin < 35 g/L | R1: Comorbidity + Albumin < 35 g/L | 2.75 | 1.29 | 5.84 | 0.009 | 588 | 58 | Yes | 9 |
| R2: Hemoglobin < 100 g/L + Albumin < 35 g/L | R2: Hemoglobin < 100 g/L + Albumin < 35 g/L | 2.41 | 1.18 | 4.96 | 0.016 | 588 | 58 | Yes | 9 |
| R3: Hemoglobin < 100 g/L + PLT < 200 × 10⁹/L | R3: Hemoglobin < 100 g/L + PLT < 200 × 10⁹/L | 1.98 | 1.00 | 3.91 | 0.050 | 588 | 58 | Yes | 9 |
| Any of three main rules | any_main_rule_3 | 2.45 | 1.36 | 4.43 | 0.003 | 588 | 58 | Yes | 9 |
| Number of main rule-positive patterns | main_rule_1 | 1.80 | 0.84 | 3.86 | 0.132 | 588 | 58 | Yes | 10 |
| Number of main rule-positive patterns | main_rule_ge2 | 3.75 | 1.73 | 8.13 | < 0.001 | 588 | 58 | Yes | 10 |

**Note:** Firth penalization was used to reduce small-sample and separation bias.

**Supplementary Table S10.** Firth logistic evaluation of qualifying association rules.

| **Antecedent** | **Antecedent N** | **Event N** | **Support** | **Confidence** | **Lift** | **OR** | **CI lower** | **CI upper** | **P value** | **Adjusted covariates** | **Converged** | **Iterations** |
| --- | --- | --- | --- | --- | --- | --- | --- | --- | --- | --- | --- | --- |
| Advanced stage (III–IV) + Albumin < 35 g/L | 77 | 14 | 0.024 | 0.182 | 1.84 | 2.45 | 1.27 | 4.73 | 0.008 | age10, male, stage_missing, comorbidity | Yes | 9 |
| Albumin < 35 g/L | 88 | 14 | 0.024 | 0.159 | 1.61 | 2.05 | 1.07 | 3.92 | 0.030 | age10, male, stage_adv, stage_missing, comorbidity | Yes | 9 |
| Hemoglobin < 100 g/L + PLT < 200 × 10⁹/L | 76 | 12 | 0.020 | 0.158 | 1.60 | 1.98 | 1.00 | 3.91 | 0.050 | age10, male, stage_adv, stage_missing, comorbidity | Yes | 9 |
| Advanced stage (III–IV) + Comorbidity + Hemoglobin < 100 g/L | 76 | 12 | 0.020 | 0.158 | 1.60 | 1.94 | 0.97 | 3.87 | 0.060 | age10, male, stage_missing | Yes | 9 |
| Advanced stage (III–IV) + Hemoglobin < 100 g/L + Albumin < 35 g/L | 53 | 11 | 0.019 | 0.208 | 2.10 | 2.84 | 1.36 | 5.89 | 0.005 | age10, male, stage_missing, comorbidity | Yes | 9 |
| Hemoglobin < 100 g/L + Albumin < 35 g/L | 60 | 11 | 0.019 | 0.183 | 1.86 | 2.41 | 1.18 | 4.96 | 0.016 | age10, male, stage_adv, stage_missing, comorbidity | Yes | 9 |
| Advanced stage (III–IV) + Hemoglobin < 100 g/L + PLT < 200 × 10⁹/L | 66 | 11 | 0.019 | 0.167 | 1.69 | 2.10 | 1.04 | 4.26 | 0.039 | age10, male, stage_missing, comorbidity | Yes | 9 |
| Advanced stage (III–IV) + Comorbidity + Albumin < 35 g/L | 40 | 10 | 0.017 | 0.250 | 2.53 | 3.51 | 1.62 | 7.60 | 0.001 | age10, male, stage_missing | Yes | 9 |
| Comorbidity + Albumin < 35 g/L | 48 | 10 | 0.017 | 0.208 | 2.11 | 2.75 | 1.29 | 5.84 | 0.009 | age10, male, stage_adv, stage_missing | Yes | 9 |
| Male sex + Advanced stage (III–IV) + Albumin < 35 g/L | 54 | 9 | 0.015 | 0.167 | 1.69 | 2.14 | 0.99 | 4.64 | 0.053 | age10, stage_missing, comorbidity | Yes | 9 |
| Male sex + Hemoglobin < 100 g/L + PLT < 200 × 10⁹/L | 55 | 9 | 0.015 | 0.164 | 1.66 | 2.01 | 0.94 | 4.31 | 0.072 | age10, stage_adv, stage_missing, comorbidity | Yes | 9 |

**Note:** Covariates are listed in the adjusted-covariate column. CI, confidence interval; OR, odds ratio.

**Supplementary Table S11.** Observed risk by core marker burden and retained rule count.

| **Stratification** | **Stratum** | **Outcome** | **N** | **Events** | **Rate** | **95% CI lower** | **95% CI upper** | **Rate, % (95% CI)** |
| --- | --- | --- | --- | --- | --- | --- | --- | --- |
| Core risk-marker burden | 0 | Hematological readmission | 263 | 13 | 0.049 | 0.029 | 0.083 | 4.9% (2.9–8.3) |
| Core risk-marker burden | 1 | Hematological readmission | 268 | 35 | 0.131 | 0.095 | 0.176 | 13.1% (9.5–17.6) |
| Core risk-marker burden | ≥ 2 | Hematological readmission | 57 | 10 | 0.175 | 0.098 | 0.294 | 17.5% (9.8–29.4) |
| Core risk-marker burden | 0 | All-cause unplanned readmission | 263 | 19 | 0.072 | 0.047 | 0.110 | 7.2% (4.7–11.0) |
| Core risk-marker burden | 1 | All-cause unplanned readmission | 268 | 46 | 0.172 | 0.131 | 0.221 | 17.2% (13.1–22.1) |
| Core risk-marker burden | ≥ 2 | All-cause unplanned readmission | 57 | 11 | 0.193 | 0.111 | 0.313 | 19.3% (11.1–31.3) |
| Rule-positive count | 0 | Hematological readmission | 471 | 38 | 0.081 | 0.059 | 0.109 | 8.1% (5.9–10.9) |
| Rule-positive count | 1 | Hematological readmission | 70 | 9 | 0.129 | 0.069 | 0.227 | 12.9% (6.9–22.7) |
| Rule-positive count | ≥ 2 | Hematological readmission | 47 | 11 | 0.234 | 0.136 | 0.372 | 23.4% (13.6–37.2) |
| Rule-positive count | 0 | All-cause unplanned readmission | 471 | 54 | 0.115 | 0.089 | 0.147 | 11.5% (8.9–14.7) |
| Rule-positive count | 1 | All-cause unplanned readmission | 70 | 10 | 0.143 | 0.079 | 0.243 | 14.3% (7.9–24.3) |
| Rule-positive count | ≥ 2 | All-cause unplanned readmission | 47 | 12 | 0.255 | 0.153 | 0.395 | 25.5% (15.3–39.5) |

**Note:** Rates are shown with Wilson 95% confidence intervals where applicable.

**Supplementary Table S12.** Apparent discrimination and Brier score for selected comparator and marker-grouping models.

| **Model** | **N** | **Events** | **Apparent AUC (95% CI)** | **Apparent Brier score** | **Mean predicted risk** |
| --- | --- | --- | --- | --- | --- |
| Clinical comparator | 588 | 58 | 0.537 (0.454–0.614) | 0.0886 | 0.099 |
| Comparator + all 3 binary core markers | 588 | 58 | 0.673 (0.608–0.734) | 0.0864 | 0.099 |
| Comparator + marker-burden strata | 588 | 58 | 0.659 (0.596–0.720) | 0.0869 | 0.099 |
| Comparator + rule-count strata | 588 | 58 | 0.638 (0.566–0.708) | 0.0870 | 0.099 |

**Note:** Probabilities used FLIC. The observed primary-event risk was 0.099. Detailed performance and optimism-corrected estimates are reported in Supplementary Table S19. AUC, area under the receiver operating characteristic curve; CI, confidence interval; FLIC, Firth's logistic regression with intercept correction.

**Supplementary Table S13.** Prespecified sensitivity analyses using Firth logistic regression.

| **Model exposure** | **Term** | **OR** | **CI lower** | **CI upper** | **P value** | **N** | **Events** | **Converged** | **Iterations** |
| --- | --- | --- | --- | --- | --- | --- | --- | --- | --- |
| Primary: core risk-marker burden, hematological readmission | core_marker_1 | 2.86 | 1.50 | 5.46 | 0.001 | 588 | 58 | Yes | 10 |
| Primary: core risk-marker burden, hematological readmission | core_marker_ge2 | 4.33 | 1.82 | 10.30 | < 0.001 | 588 | 58 | Yes | 10 |
| Primary: rule-count, hematological readmission | main_rule_1 | 1.80 | 0.84 | 3.86 | 0.132 | 588 | 58 | Yes | 10 |
| Primary: rule-count, hematological readmission | main_rule_ge2 | 3.75 | 1.73 | 8.13 | < 0.001 | 588 | 58 | Yes | 10 |
| Sensitivity: excluding planned admissions, core risk-marker burden | core_marker_1 | 2.73 | 1.43 | 5.22 | 0.002 | 551 | 58 | Yes | 10 |
| Sensitivity: excluding planned admissions, core risk-marker burden | core_marker_ge2 | 4.12 | 1.72 | 9.85 | 0.001 | 551 | 58 | Yes | 10 |
| Sensitivity: excluding planned admissions, rule count | main_rule_1 | 1.69 | 0.78 | 3.63 | 0.181 | 551 | 58 | Yes | 10 |
| Sensitivity: excluding planned admissions, rule count | main_rule_ge2 | 3.46 | 1.60 | 7.51 | 0.002 | 551 | 58 | Yes | 10 |
| Sensitivity: PLT < 150 × 10⁹/L threshold core risk-marker burden | core150_marker_1 | 1.71 | 0.93 | 3.13 | 0.081 | 588 | 58 | Yes | 10 |
| Sensitivity: PLT < 150 × 10⁹/L threshold core risk-marker burden | core150_marker_ge2 | 3.40 | 1.45 | 7.98 | 0.005 | 588 | 58 | Yes | 10 |
| Sensitivity: all-cause unplanned readmission, core risk-marker burden | core_marker_1 | 2.65 | 1.52 | 4.61 | < 0.001 | 588 | 76 | Yes | 10 |
| Sensitivity: all-cause unplanned readmission, core risk-marker burden | core_marker_ge2 | 3.18 | 1.43 | 7.05 | 0.004 | 588 | 76 | Yes | 10 |
| Sensitivity: all-cause unplanned readmission, rule count | main_rule_1 | 1.34 | 0.65 | 2.75 | 0.425 | 588 | 76 | Yes | 9 |
| Sensitivity: all-cause unplanned readmission, rule count | main_rule_ge2 | 2.83 | 1.36 | 5.86 | 0.005 | 588 | 76 | Yes | 9 |
| Sensitivity: stage-complete sample, core risk-marker burden | core_marker_1 | 2.79 | 1.46 | 5.34 | 0.002 | 578 | 57 | Yes | 7 |
| Sensitivity: stage-complete sample, core risk-marker burden | core_marker_ge2 | 4.44 | 1.86 | 10.61 | < 0.001 | 578 | 57 | Yes | 7 |
| Sensitivity: center-adjusted Firth, core risk-marker burden | core_marker_1 | 2.89 | 1.52 | 5.49 | 0.001 | 588 | 58 | Yes | 9 |
| Sensitivity: center-adjusted Firth, core risk-marker burden | core_marker_ge2 | 4.17 | 1.75 | 9.91 | 0.001 | 588 | 58 | Yes | 9 |
| Sensitivity: center-adjusted Firth, rule count | main_rule_1 | 1.98 | 0.92 | 4.28 | 0.083 | 588 | 58 | Yes | 9 |
| Sensitivity: center-adjusted Firth, rule count | main_rule_ge2 | 3.78 | 1.74 | 8.20 | < 0.001 | 588 | 58 | Yes | 9 |
| Sensitivity: excluding zero-event center, core risk-marker burden | core_marker_1 | 2.90 | 1.52 | 5.52 | 0.001 | 571 | 58 | Yes | 10 |
| Sensitivity: excluding zero-event center, core risk-marker burden | core_marker_ge2 | 4.31 | 1.80 | 10.31 | 0.001 | 571 | 58 | Yes | 10 |
| Sensitivity: excluding zero-event center, rule count | main_rule_1 | 1.79 | 0.83 | 3.87 | 0.136 | 571 | 58 | Yes | 10 |
| Sensitivity: excluding zero-event center, rule count | main_rule_ge2 | 3.77 | 1.73 | 8.20 | < 0.001 | 571 | 58 | Yes | 10 |

**Note:** Sensitivity analyses assessed alternative thresholds, outcomes, center specifications, and exclusion of planned treatment-continuation admissions. The planned-admission exclusion model used N = 551 because 37 of the 38 planned admissions occurred in the 588-patient primary analytic sample.

**Supplementary Table S14.** Data dictionary and variable definitions.

| **Variable** | **Definition** |
| --- | --- |
| hem_readmit | 1 if the chronologically first readmission within 30 days after index discharge was unplanned and its reconciled principal-reason category was hematological toxicity/myelosuppression; otherwise 0 |
| unplanned30 | 1 if the chronologically first readmission within 30 days after index discharge was classified as unplanned; otherwise 0 |
| planned_admission | 1 if the chronologically first readmission within 30 days after index discharge was for planned treatment continuation; otherwise 0 |
| ANC_low_1_5 | 1 if absolute neutrophil count measured during the 7-calendar-day window ending on and including index discharge was < 1.5 × 10⁹/L; 0 otherwise |
| PLT_low_200 | 1 if platelet count measured during the 7-calendar-day window ending on and including index discharge was < 200 × 10⁹/L; 0 otherwise |
| PLT_low_150 | 1 if platelet count measured during the 7-calendar-day window ending on and including index discharge was < 150 × 10⁹/L; 0 otherwise; used as a sensitivity threshold |
| ALB_low_35 | 1 if albumin measured during the 7-calendar-day window ending on and including index discharge was < 35 g/L; 0 otherwise |
| Hb_low_100 | 1 if hemoglobin measured during the 7-calendar-day window ending on and including index discharge was < 100 g/L; 0 otherwise |
| BMI_under_18_5 | 1 if BMI < 18.5 kg/m² and 0 if BMI ≥ 18.5 kg/m², using the most recent BMI measurement within the 7-calendar-day window ending on and including index discharge; missing BMI remained unclassified and was excluded from BMI-specific descriptive comparisons |
| core_marker_count | Number of ANC_low_1_5, PLT_low_200, and ALB_low_35 core markers present |
| core_marker_group | Core risk-marker group coded as 0, 1, or ≥ 2 markers |
| main_rule_count_3 | Number of the three main pairwise rules satisfied |
| main_rule_group_3 | Main rule-positive group coded as 0, 1, or ≥ 2 rules satisfied |
| stage_missing | 1 if the source clinical-stage field was missing; otherwise 0 |
| stage_adv | 1 if clinical stage was III–IV; missing stage was coded as 0 and flagged by stage_missing |
| age10 | Age in years divided by 10 for adjusted Firth regression models |
| male | 1 for male sex and 0 for female sex |
| comorbidity_present | 1 if any recorded chronic comorbidity was present in the index-admission medical record; otherwise 0 |
| hospital_center | Participating hospital identifier; included in sensitivity models rather than the primary model because one center had no hematological readmission events |
| rule_positive_indicator | Binary variable equal to 1 when all antecedent items in a specified association rule were present; otherwise 0 |
| hem_readmit_time | Days from index discharge to the chronologically first readmission within 30 days for patients whose first readmission was unplanned and classified as hematological toxicity-related; used only to generate the descriptive cumulative readmission figure. |
| measurement_window | All laboratory, BMI, and threshold variables used the most recent measurement within the 7-calendar-day window ending on and including index discharge during the index hospitalization |
| any_chemotherapy | 1 if at least one chemotherapy administration before index discharge was documented; 0 otherwise; discordant summary fields were reconciled against dated administrations and documented refusal |
| induction_chemotherapy | 1 for chemotherapy administered before radiotherapy; 0 otherwise |
| concurrent_chemotherapy | 1 for chemotherapy administered during radiotherapy; 0 otherwise |
| predischarge_adjuvant | 1 for chemotherapy administered after radiotherapy but before index discharge; 0 otherwise; treatment first administered after discharge was excluded |
| chemotherapy_cycles | Cumulative administered cycles in the current treatment course through index discharge; patients with no documented chemotherapy administration before discharge were coded as 0 cycles |
| chemo_to_discharge_days | Calendar days from the most recent chemotherapy administration to index discharge among chemotherapy recipients |
| supportive_care_7d | Six separate exposure variables—G-CSF, systemic antimicrobial treatment, nutritional support, red-cell transfusion, platelet transfusion, and human albumin infusion—during the 7-calendar-day window ending on and including index discharge. Each was coded 1 for documented administration and 0 for no administration in an accessible executed-order history; an inaccessible history was coded missing |
| clinical_comparator | Age per 10 years, male sex, stage III–IV, missing-stage indicator, and comorbidity |
| FLIC_probability | Predicted probability from Firth's logistic regression with intercept correction; used for Brier score, calibration, and decision-curve analysis |

**Note:** Laboratory and anthropometric variables used the measurement closest to discharge within the 7-calendar-day window ending on and including index discharge.

**Supplementary Table S15.** Regression coding, method-use conditions, and diagnostics.

| **Component** | **Implementation or coding** | **Diagnostic result** | **Interpretation** |
| --- | --- | --- | --- |
| Analysis unit | One row per patient; only the first eligible index hospitalization was retained. | Full cohort N = 607; primary complete-case analytic sample N = 588. | Observation independence was addressed at the patient-record level. |
| Primary outcome | Chronologically first readmission within 30 days after index discharge classified as unplanned with hematological toxicity/myelosuppression as the reconciled principal reason; coded 1 = yes and 0 = no. | Events = 58 in the primary analytic sample. | Outcome was a post-discharge hospital-utilization event, not a laboratory value. |
| Core risk markers | ANC < 1.5 × 10⁹/L; PLT < 200 × 10⁹/L; albumin < 35 g/L; each coded 1 = marker positive and 0 = marker negative. | Marker count grouped as 0, 1, or ≥ 2 markers. | Grouping limited model complexity and supported clinical interpretability. |
| Adjustment covariates | Age per 10-year increase; male sex; stage III–IV; missing-stage indicator; comorbidity present. | Categorical variables used dummy coding with prespecified marker-negative or absent categories as references. | Coding was prespecified and avoids mixing stage components with overall stage. |
| Collinearity diagnostics | Variance inflation factors from auxiliary regression models for the adjusted core-marker model. | Age 1.03; male sex 1.02; stage III–IV 1.20; missing-stage indicator 1.21; comorbidity 1.03; ANC marker 1.02; PLT marker 1.02; albumin marker 1.03. | All VIFs were far below conventional concern thresholds, supporting inclusion in adjusted models. |
| Sparse-data handling | Firth penalized logistic regression with 95% confidence intervals. | Several rule-defined subgroups had small event counts; the primary outcome had 58 events. | Firth estimation was used to reduce small-sample and separation bias. |
| Categorical comparisons | Pearson χ² test when expected counts were adequate; Fisher exact test for sparse cells. | Sparse-cell handling was applied as needed in baseline comparisons. | This avoids relying on large-sample chi-square approximations in sparse tables. |
| Association rule mining | Outcome fixed as consequent; minimum co-occurrence support 1.5%, confidence 15%, lift 1.5, antecedent length ≤ 3, and ≥ 9 events per retained rule. | Rules below the event-count threshold were not used as main rules. | Prespecified thresholds reduce post hoc selection and improve reproducibility. |

**Note:** ANC, absolute neutrophil count; PLT, platelet count; VIF, variance inflation factor.

**Supplementary Table S16.** Chemotherapy and supportive-care characteristics in the primary analytic sample.

| **Variable** | **Evaluable N** | **Distribution** | **Outcome summary** |
| --- | --- | --- | --- |
| Any chemotherapy before discharge | 588 | 578 (98.3) | 57/578 (9.9) |
| Induction chemotherapy | 588 | 540 (91.8) | 54/540 (10.0) |
| Concurrent chemotherapy | 588 | 415 (70.6) | 40/415 (9.6) |
| Pre-discharge adjuvant chemotherapy | 588 | 1 (0.2) | 0/1 (0.0) |
| Cumulative administered cycles | 588 | Median 5 (IQR 3–6) | Median 5.0 (IQR 3.3–6.0) |
| Last chemotherapy-to-discharge interval | 578 | Median 20 days (IQR 7–52) | Median 22 days (IQR 8–61) |
| Primary platinum agent: cisplatin | 588 | 409 (69.6) | 35/409 (8.6) |
| Primary platinum agent: nedaplatin | 588 | 152 (25.9) | 18/152 (11.8) |
| Primary platinum agent: carboplatin | 588 | 12 (2.0) | 1/12 (8.3) |
| Primary platinum agent: lobaplatin | 588 | 3 (0.5) | 2/3 (66.7) |
| No primary platinum agent/other | 588 | 12 (2.0) | 2/12 (16.7) |
| Induction regimen: nab-paclitaxel + cisplatin | 588 | 103 (17.5) | 14/103 (13.6) |
| Induction regimen: docetaxel + nedaplatin | 588 | 98 (16.7) | 13/98 (13.3) |
| Induction regimen: docetaxel + cisplatin | 588 | 89 (15.1) | 7/89 (7.9) |
| Other/no documented induction regimen | 588 | 298 (50.7) | 24/298 (8.1) |
| Concurrent regimen: weekly cisplatin | 588 | 185 (31.5) | 22/185 (11.9) |
| Concurrent regimen: three-weekly cisplatin | 588 | 175 (29.8) | 9/175 (5.1) |
| Concurrent regimen: weekly nedaplatin | 588 | 21 (3.6) | 4/21 (19.0) |
| Other/no documented concurrent regimen | 588 | 207 (35.2) | 23/207 (11.1) |
| G-CSF during the 7-calendar-day window ending on and including index discharge | 520 | 259 (49.8) | 32/259 (12.4) |
| Systemic antimicrobial treatment | 520 | 98 (18.8) | 12/98 (12.2) |
| Nutritional support | 520 | 105 (20.2) | 21/105 (20.0) |
| Red-cell transfusion | 520 | 1 (0.2) | 0/1 (0.0) |
| Platelet transfusion | 520 | 1 (0.2) | 0/1 (0.0) |
| Human albumin infusion | 518 | 6 (1.2) | 3/6 (50.0) |

**Note:** Percentages use the stated evaluable denominator. For categorical variables, the outcome summary is hematological events/category total (%); for continuous variables, it is the median (IQR) among patients with hematological readmission. Platinum categories and the three most common exact induction and concurrent regimen labels are shown descriptively; remaining labels and patients without the corresponding regimen were combined. Treatment first administered after index discharge was not a baseline exposure. Internally discordant summary fields were reconciled against dated administration records and documented treatment refusal.

**Supplementary Table S17.** Chemotherapy and supportive-care sensitivity models.

| **Model** | **N** | **Events** | **1 marker vs 0, OR (95% CI)** | **≥ 2 markers vs 0, OR (95% CI)** |
| --- | --- | --- | --- | --- |
| Primary marker-burden model | 588 | 58 | 2.86 (1.50–5.46); P = 0.001 | 4.33 (1.82–10.30); P < 0.001 |
| C1 chemotherapy-adjusted | 588 | 58 | 2.93 (1.52–5.64); P = 0.001 | 4.55 (1.88–11.02); P < 0.001 |
| C2 chemotherapy recipients | 578 | 57 | 2.84 (1.47–5.49); P = 0.002 | 4.51 (1.85–11.03); P < 0.001 |
| S0 supportive-care-evaluable subset | 520 | 57 | 2.64 (1.37–5.07); P = 0.004 | 4.27 (1.77–10.25); P = 0.001 |
| S1 supportive-care adjusted | 520 | 57 | 2.59 (1.34–5.02); P = 0.005 | 3.47 (1.38–8.76); P = 0.008 |
| No concurrent chemotherapy | 173 | 18 | 3.01 (0.94–9.68); P = 0.064 | 9.63 (1.85–50.20); P = 0.007 |
| Concurrent chemotherapy | 415 | 40 | 2.70 (1.24–5.88); P = 0.012 | 3.38 (1.22–9.41); P = 0.020 |

**Note:** All models used Firth logistic regression and adjusted for age, sex, stage, missing stage, and comorbidity. C1 additionally included cumulative cycles (per one additional administered cycle) and concurrent chemotherapy; C2 included cycles and the chemotherapy-to-discharge interval among chemotherapy recipients; S1 added G-CSF, antimicrobial treatment, and nutritional support. Additional covariate estimates were: C1 concurrent chemotherapy, OR 0.84 (95% CI 0.42–1.71; P = 0.639), and cycles, OR 0.98 (0.85–1.13; P = 0.791); C2 cycles, OR 0.98 (0.85–1.12; P = 0.726), and chemotherapy-to-discharge interval, OR 1.002 per day (0.996–1.007; P = 0.564); S1 G-CSF, OR 1.01 (0.57–1.80; P = 0.973), systemic antimicrobial treatment, OR 1.02 (0.51–2.04; P = 0.949), and nutritional support, OR 2.41 (1.33–4.38; P = 0.004). Supportive-care coefficients may reflect confounding by indication.

**Supplementary Table S18.** Conventional-threshold and continuous-marker sensitivity analyses.

| **Analysis/term** | **Adjusted OR (95% CI)** | **P value** |
| --- | --- | --- |
| Marker burden with PLT < 150 × 10⁹/L: 1 vs 0 | 1.71 (0.93–3.13) | 0.081 |
| Marker burden with PLT < 150 × 10⁹/L: ≥ 2 vs 0 | 3.40 (1.45–7.98) | 0.005 |
| ANC per 1 × 10⁹/L lower (single-marker model) | 0.97 (0.93–1.02) | 0.273 |
| PLT per 50 × 10⁹/L lower (single-marker model) | 1.16 (0.96–1.41) | 0.125 |
| Albumin per 5 g/L lower (single-marker model) | 1.32 (0.95–1.84) | 0.097 |
| ANC per 1 × 10⁹/L lower (joint continuous model) | 0.97 (0.92–1.02) | 0.222 |
| PLT per 50 × 10⁹/L lower (joint continuous model) | 1.15 (0.95–1.39) | 0.147 |
| Albumin per 5 g/L lower (joint continuous model) | 1.29 (0.92–1.80) | 0.136 |

**Note:** All models adjusted for age, sex, stage, missing stage, and comorbidity. PLT < 200 × 10⁹/L was a prespecified exploratory marker, not a clinical thrombocytopenia threshold. No outcome-guided cut-point selection was conducted.

**Supplementary Table S19.** Detailed internal performance of FLIC models.

| **Model** | **Apparent AUC** | **Corrected AUC** | **Apparent Brier** | **Corrected Brier** | **Apparent calibration-in-the-large** | **Apparent calibration intercept** | **Apparent slope** | **Corrected slope** |
| --- | --- | --- | --- | --- | --- | --- | --- | --- |
| Clinical comparator | 0.537 | 0.476 | 0.0886 | 0.0903 | 0.000 | −0.246 | 0.888 | 0.166 |
| Comparator + continuous ANC | 0.562 | 0.495 | 0.0885 | 0.0906 | 0.000 | −0.237 | 0.892 | 0.228 |
| Comparator + continuous PLT | 0.619 | 0.557 | 0.0882 | 0.0903 | 0.000 | −0.064 | 0.971 | 0.393 |
| Comparator + continuous albumin | 0.582 | 0.522 | 0.0883 | 0.0904 | 0.000 | −0.088 | 0.959 | 0.432 |
| Comparator + all 3 continuous measures | 0.626 | 0.555 | 0.0879 | 0.0909 | 0.000 | −0.049 | 0.977 | 0.468 |
| Comparator + all 3 binary core markers | 0.673 | 0.624 | 0.0864 | 0.0898 | 0.000 | −0.015 | 0.993 | 0.717 |
| Comparator + marker-burden strata | 0.659 | 0.615 | 0.0869 | 0.0896 | 0.000 | 0.008 | 1.004 | 0.732 |
| Comparator + rule-count strata | 0.638 | 0.585 | 0.0870 | 0.0899 | 0.000 | −0.039 | 0.982 | 0.674 |

**Note:** FLIC forces the apparent mean predicted risk to equal observed risk, so calibration-in-the-large, calculated with the slope fixed at 1, is 0 subject to rounding. The separate calibration intercept was estimated jointly with the calibration slope. Corrected values were based on 1,000 bootstrap refits. The rule-count estimate is conditional because rule selection was not repeated in each bootstrap sample. AUC, area under the receiver operating characteristic curve; FLIC, Firth's logistic regression with intercept correction.

**Supplementary Table S20.** Outcome-reason dictionary and final mutually exclusive counts in the 607-patient cohort.

| **Mutually exclusive category** | **Operational description** | **N** | **Analysis status** |
| --- | --- | --- | --- |
| Hematological toxicity/myelosuppression | Myelosuppression, hematological toxicity, or clinically equivalent cytopenic/toxicity category recorded as principal inpatient reason | 58 | Primary event |
| Infection/fever/pulmonary complication | Acute infection, fever, or pulmonary complication | 6 | All-cause event only |
| Radiation mucositis/pharyngeal symptom | Radiation mucositis or acute pharyngeal symptom requiring admission | 6 | All-cause event only |
| Nutritional/gastrointestinal problem | Acute nutritional or gastrointestinal reason | 3 | All-cause event only |
| Other acute/cardiovascular/cerebrovascular | Other acute principal reason, including cardiovascular/cerebrovascular | 2 | All-cause event only |
| Skin/radiation dermatitis | Acute skin or radiation dermatitis admission | 1 | All-cause event only |
| Other/unclear acute cause | Unplanned admission with an acute but otherwise unclassifiable principal reason | 1 | All-cause event only |
| Planned treatment continuation | Admission explicitly documented for planned treatment continuation | 38 | Non-event |
| No recorded 30-day readmission | No inpatient readmission was recorded within 30 days in participating-hospital records | 492 | Non-event |

**Note:** For patients with more than one readmission within 30 days after index discharge, only the chronologically first readmission was used to determine patient-level outcome status. If that admission was for planned treatment continuation, it was classified as a non-event, and subsequent readmissions did not redefine the outcome. If it was unplanned, its principal documented reason determined the mutually exclusive category; no severity-based or cause-priority hierarchy was applied. Two researchers independently assigned planned versus unplanned status and the principal reason using the shared dictionary; disagreements were resolved by joint review of the source records and consensus. Predictor values and derived marker/rule status were not used for classification. Only the reconciled classification was retained, so inter-rater agreement and Cohen's κ could not be estimated retrospectively.
